# Bacteria‐Mediated Tumor‐Targeting Delivery of Multienzyme‐Mimicking Covalent Organic Frameworks Promoting Pyroptosis for Combinatorial Sono‐Catalytic Immunotherapy

**DOI:** 10.1002/advs.202407133

**Published:** 2024-11-04

**Authors:** Yunyun Liu, Lihua Xiang, Yitong Li, Shen Zhang, Ying Zhang, Hui Shi, Hui Liu, Dou Du, Bangguo Zhou, Beibei Ye, Shaoyue Li, Haohao Yin, Huixiong Xu, Yifeng Zhang

**Affiliations:** ^1^ Department of Medical Ultrasound Center of Minimally Invasive Treatment for Tumor Shanghai Tenth People's Hospital School of Medicine Tongji University Shanghai 200072 P. R. China; ^2^ Ultrasound Research and Education Institute Clinical Research Center for Interventional Medicine Shanghai Tenth People's Hospital School of Medicine Tongji University Shanghai 200072 P. R. China; ^3^ Shanghai Engineering Research Center of Ultrasound Diagnosis and Treatment Shanghai 200072 P. R. China; ^4^ Department of Ultrasound Zhongshan Hospital Institute of Ultrasound in Medicine and Engineering Fudan University Shanghai 200032 P. R. China

**Keywords:** immunotherapy, multienzyme mimicking, pyroptosis, tumor‐targeting delivery, ultrasound

## Abstract

Pyroptosis, an inflammatory cell death, has attracted great attention for potentiating a strong immune response against tumor cells. However, developing powerful pyroptosis inducers and then activating specific pyroptosis still remains challenging. Herein, a PEG‐CuP‐COF@∆St nanosystem is rationally designed, consisting of PEG‐CuP‐COF nanozyme pyroptosis inducers and tumor‐targeting bacteria of the Salmonella Typhimurium strain VNP20009 (ΔSt), with an affinity for the tumor hypoxic microenvironment. The PEG‐CuP‐COF nanozymes possessed excellent sonodynamic performance and multienzyme‐mimicking activities to generate reactive oxygen species (ROS) and then induce potent pyroptosis. The superoxide dismutase‐ and peroxidase‐mimicking activities of PEG‐CuP‐COF catalytically produced hydrogen peroxide (H_2_O_2_) and hydroxyl radicals (•OH) which have important value in triggering acute inflammatory responses and pyroptosis. Moreover, PEG‐CuP‐COF showed outstanding glutathione peroxidase‐mimicking activities, impairing the antioxidant defense in tumor cells and enhancing sonodynamic efficiency by making them more vulnerable to ROS‐induced damage. During in vivo studies, PEG‐CuP‐COF@∆St nanosystem with its self‐driven property exhibited impressive tumor‐targeting capability and activated Caspase‐3/gasdermin E‐dependent pyroptosis to inhibit tumor growth. More importantly, it induced a powerful immune memory effect to prevent bone metastasis. In summary, this study introduces an innovative approach for combinatorial sono‐catalytic immunotherapy using bacteria‐mediated tumor‐targeting delivery of nanozymes as specific pyroptosis inducers.

## Graphical abstract

This study presents a novel PEG‐CuP‐COF@∆St nanosystem that consists of PEG‐CuP‐COF nanozyme pyroptosis inducers and tumor‐targeting bacteria of the Salmonella Typhimurium strain VNP20009 (ΔSt). The PEG‐CuP‐COF@∆St nanosystem can specifically induce pyroptosis in tumor cells. This strategy inhibits both primary and distant tumors and prevents bone metastasis, providing an innovative paradigm for combinatorial sono‐catalytic immunotherapy.

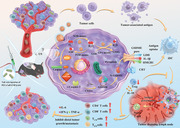

## 1 Introduction

Pyroptosis, an inflammatory cell death, has gained great attention as a novel kind of immunogenic cell death (ICD).^[^ ^1^ ^]^ Unlike the non‐inflammatory processes of apoptosis, pyroptosis is distinguished by the formation of transmembrane pores, cell swelling, plasma membrane rupture, and the release of pro‐inflammatory cytokines and damage‐associated molecular patterns (DAMPs), which can stimulate a strong immune response against tumor cells.^[^ ^2^, ^3^, ^4^, ^5^ ^]^ Given the limited therapeutic efficacy of apoptosis due to apoptosis resistance in tumor cells, pyroptosis has been extensively studied as an alternative strategy for combating cancers and is proven to be an effective method due to its excellent ability to prime antitumor immune responses.^[^ ^6^, ^7^, ^8^ ^]^ Moreover, research has shown that pyroptosis is involved in regulating tumor proliferation, differentiation, invasiveness, and metastasis.^[^ ^9^, ^10^, ^11^ ^]^ Therefore, the development of potent pyroptosis inducers is crucial for advancing antitumor immunotherapy.

Current evidence suggests that reactive oxygen species (ROS) can induce pyroptosis and the precise mechanism has been validated, wherein ROS acts as a crucial inflammasome‐activating signal or causes oxidative damage to the mitochondria and cell membrane.^[^ ^12^, ^13^, ^14^, ^15^ ^]^ For example, hydroxyl radicals (•OH), as the most toxic ROS, can effectively induce pyroptosis while not relying on the penetration depth or oxygen levels.^[^ ^16^, ^17^ ^]^ Consequently, ROS‐induced pyroptosis holds promise as a novel target for antitumor immunotherapy. Sonodynamic therapy (SDT), a non‐invasive therapeutic approach, utilizes ultrasound (US) irradiation to activate sonosensitizers like porphyrin, thereby generating ROS.^[^ ^18^, ^19^, ^20^ ^]^ Crucially, whether from internal or external origins, the increased buildup of ROS within tumor cells stemming from an inability to promptly mitigate ROS levels, renders them more vulnerable to ROS‐induced cytotoxicity and pyroptosis compared to normal cells, thus selectively exerting anticancer effects.^[^ ^21^ ^]^ Therefore, SDT shows potential for triggering pyroptosis and addressing drawbacks of traditional tumor treatments by providing improved specificity and fewer side effects. Nonetheless, ROS produced by SDT typically exhibits transient and inadequate characteristics for eliciting robust pyroptosis.

In order to produce ROS efficiently, nanoparticles have been engineered with enzyme‐mimicking characteristics and widely used in tumor‐catalytic therapy, leading to the emerging field of nanozymes.^[^ ^22^, ^23^, ^24^ ^]^ In comparison to normal nanoparticles lacking enzyme activities, nanozymes exhibit significant capability in ROS generation, thereby triggering pyroptotic cell death in antitumor treatments. Among various nanozymes, metallic copper (Cu)‐based nanozymes are commonly applied in tumor therapy for their superior catalytic performance and certain biological stability, which may not be as effectively achieved with other covalent metals like manganese (Mn) and zinc (Zn)‐based systems.^[^ ^25^ ^]^ Owing to its unique electronic configuration, Cu‐based nanozymes can participate in diverse catalytic reactions, including superoxide dismutase (SOD), peroxidase (POD), glutathione peroxidase (GPx)‐mimicking activities, etc, making Cu a more effective catalyst for reactions involving ROS generation.^[^ ^25^, ^26^ ^]^ Hence, copper ions are chosen to coordinate within porphyrin units for synthesizing nanozymes to improve SDT performance and ROS‐induced pyroptosis. However, ROS catalytically generated by nanozymes may inadvertently damage the chemical structure of porphyrin due to the irregular arrangement of molecules, resulting in suboptimal therapeutic outcomes.^[^ ^27^, ^28^ ^]^ Therefore, it is necessary to enhance the regularity of spatial distribution and stability of chemical structure of molecules for improving the performance of Cu/porphyrin‐based nanozymes. Covalent organic frameworks (COFs), with well‐defined structures and ease of functionalization, provide an excellent strategy for constructing nanozymes.^[^ ^29^, ^30^ ^]^ Studies have demonstrated that porphyrin molecules display a uniform distribution within COFs, shielding them from ROS‐induced damage owing to the robust framework structure.^[^ ^26^, ^28^, ^31^ ^]^ Furthermore, the active sites of Cu ions in nanozymes can be integrated into the backbone of COFs, enhancing selectivity and achieving ultrahigh activity.^[^ ^32^ ^]^ Notably, these COF‐based nanozymes might inadvertently affect normal cells due to their inability to target tumor specifically, leading to unsatisfactory therapeutic effect. Therefore, how to achieve tumor‐targeting delivery and then induce specific pyroptosis in tumor cells may be a research hotspot.

Recently, there has been considerable interest in bacteria‐mediated tumor‐targeting delivery systems due to their remarkable ability to overcome physiological hurdles, including tissue penetration and tumor hypoxia, etc.^[^ ^33^ ^]^ As tumor growth relies heavily on angiogenesis and creates a hypoxic microenvironment, the development of genetically engineered bacteria with an affinity for hypoxia presents a promising avenue for cancer treatment.^[^ ^34^, ^35^ ^]^ Attenuated Salmonella Typhimurium strain VNP20009 (ΔSt), as the only Salmonella strain to be evaluated in a clinical trial, is proven to be a potential tumor‐targeting bacterium for its ability to preferentially accumulate and replicate in hypoxic areas of solid tumors with its self‐driven property, thereby significantly minimizing the adverse effects on normal tissues.^[^ ^36^ ^]^ Research has indicated that ΔSt can enable efficient delivery and accumulation of nanoparticles in tumor sites, and further result in much‐inhibited growth of tumors and activate a long‐term immune memory effect when combined with immune checkpoint therapy.^[^ ^37^, ^38^ ^]^ Moreover, ΔSt itself also exhibits excellent bio‐safety.^[^ ^39^ ^]^ Considering these findings, ΔSt bacteria hold substantial practical value as carriers in tumor‐targeting delivery system for nanotherapeutic strategy.

**Figure 1:**
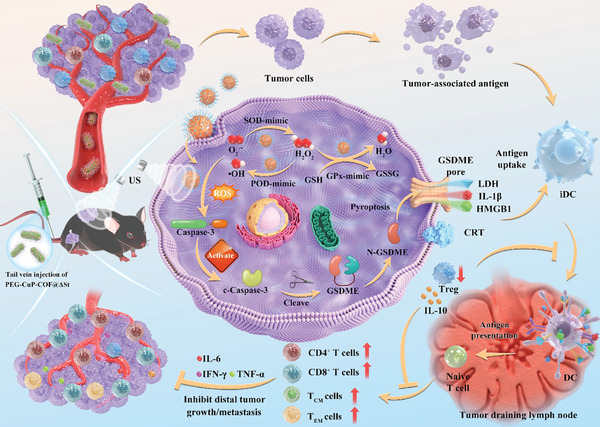
Schematic illustration of PEG‐CuP‐COF@∆St nanosystem promoting pyroptosis for combinatorial sono‐catalytic immunotherapy. The PEG‐CuP‐COF@∆St nanosystem, which consists of PEG‐CuP‐COF nanozyme pyroptosis inducers and tumor‐targeting ΔSt bacteria, was successfully designed and activated ROS‐induced pyroptosis in RM‐1 prostate cancer cells. This process was accompanied by Caspase‐3 activation, GSDME cleavage, and the release of IL‐1β, LDH, HMGB1, and CRT, ultimately leading to enhanced pyroptotic cell death, specific immune responses, and long‐term immune memory.

In the present study, we successfully designed the multienzyme‐mimicking COFs named PEG‐CuP‐COF to promote ROS‐induced pyroptosis, by integrating porphyrin molecules and copper ions into COFs structures. Besides, a novel Salmonella‐mediated tumor‐targeting delivery system of PEG‐CuP‐COF@∆St was developed (**Figure** **1**). It was proven that the PEG‐CuP‐COF exhibited excellent multienzyme‐mimicking activities. Initially, porphyrin molecules encapsulated within COFs structures produced various ROS under US irradiation in which the superoxide anion radicals (O_2_^−•^) were converted into hydrogen peroxide (H_2_O_2_) catalyzed by the SOD‐mimicking activity. Simultaneously, PEG‐CuP‐COF nanosystem exerted POD‐mimicking activity to facilitate the consumption of H_2_O_2_ to yield •OH which is the most harmful type of ROS. The PEG‐CuP‐COF also possessed remarkable GPx‐mimicking activities that employ cellular tripeptide glutathione (GSH) as a reductant to catalyze the conversion of H_2_O_2_ to H_2_O, while generating oxidized glutathione (GSSG) and depleting GSH. Through GSH depletion, PEG‐CuP‐COF nanosystem compromised the antioxidant defense in tumor cells and made them more vulnerable to ROS‐induced damage. Summarily, the multienzyme‐mimicking properties of PEG‐CuP‐COF disrupted cellular redox homeostasis, thereby improving SDT performance and ROS‐induced pyroptosis in tumor cells. During this process, the generated ROS activated Caspase‐3/gasdermin E (GSDME)‐dependent pyroptosis in RM‐1 prostate cancer cells, leading to the release of abundant DAMPs and pro‐inflammatory cytokines. In vivo studies indicated that the self‐driven PEG‐CuP‐COF@∆St nanosystem not only possessed desirable biocompatibility but also exhibited a favorable tumor‐targeting characteristic. Our results demonstrated that PEG‐CuP‐COF@∆St nanosystem effectively suppressed the progression of RM‐1 primary and distant tumors, and prevented bone metastases, indicating superiority in the activation of systemic adaptive immune response. Conclusively, these favorable results indicated effectiveness of the well‐designed PEG‐CuP‐COF@∆St nanosystem in triggering pyroptosis for combinatorial sono‐catalytic immunotherapy. Importantly, this study provides a new avenue for antitumor treatment through using bacteria‐mediated tumor‐targeting nanozymes as pyroptosis inducers.

## 2 Results

### 2.1 Synthesis and Characterizations of the Self‐Driven PEG‐CuP‐COF@∆St Nanosystem

**Figure 2:**
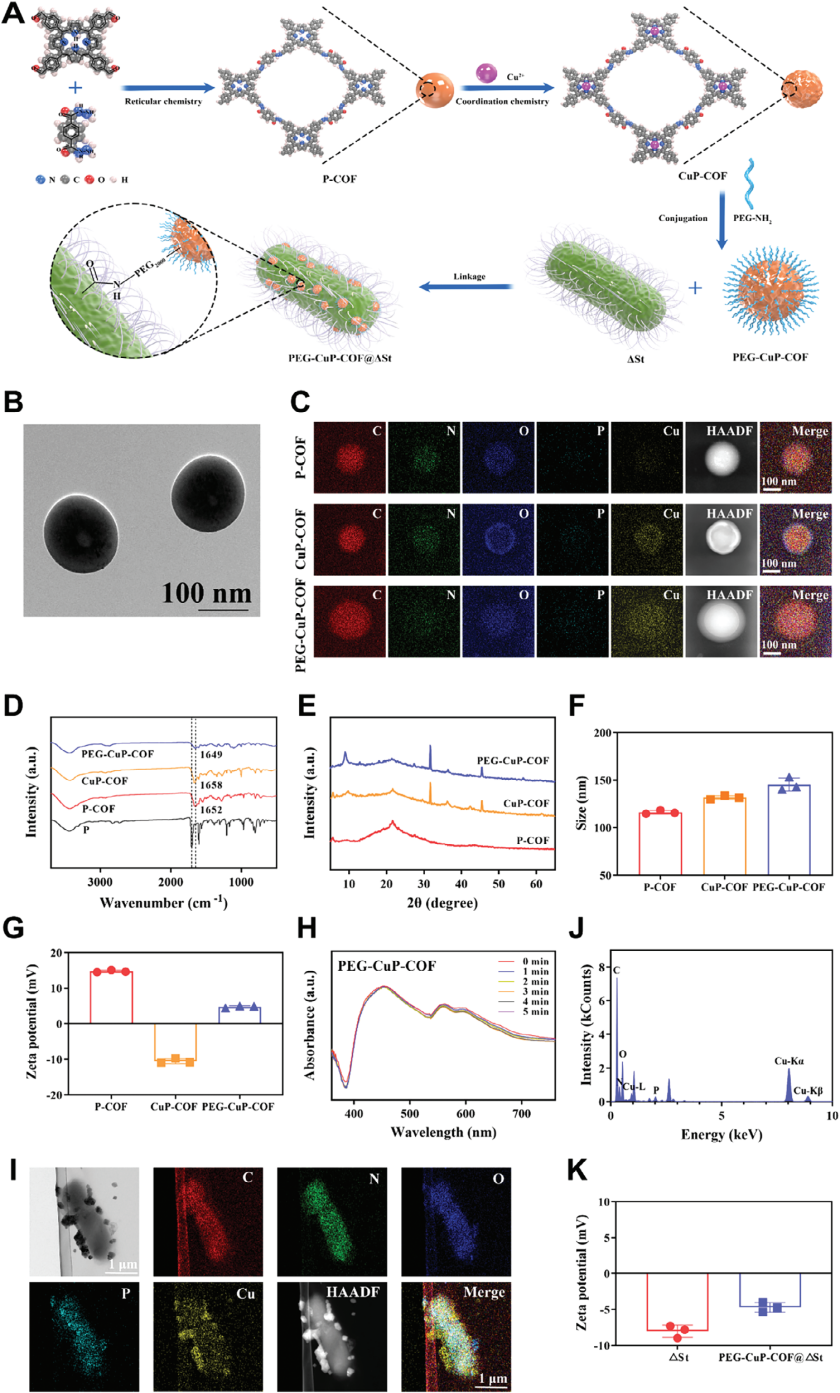
Synthesis and characterizations of PEG‐CuP‐COF and PEG‐CuP‐COF@∆St. A) Synthesis strategy to prepare PEG‐CuP‐COF and PEG‐CuP‐COF@∆St. B) Transmission electron microscopic (TEM) image of PEG‐CuP‐COF. C) Elemental mapping images (C, N, O, P, and Cu elements) of P‐COF, CuP‐COF and PEG‐CuP‐COF. D) Fourier transform infrared (FT‐IR) spectra of P, P‐COF, CuP‐COF, and PEG‐CuP‐COF. E) X‐ray diffraction (XRD) spectra of P‐COF, CuP‐COF, and PEG‐CuP‐COF. F) Particle size and G) zeta potential of P‐COF, CuP‐COF, and PEG‐CuP‐COF. H) UV–vis absorption spectra of PEG‐CuP‐COF under US irradiation for different times (1.0 MHz, 1.0 W cm^−2^, 50% duty cycle). I) Elemental mapping images (C, N, O, P, and Cu elements) and J) the corresponding energy‐dispersive X‐ray spectroscopy (EDS) elemental analysis of PEG‐CuP‐COF@∆St. K) Zeta potential of ∆St and PEG‐CuP‐COF@∆St. Data are presented as Mean ± SD.

The synthesis of PEG‐CuP‐COF and PEG‐CuP‐COF@∆St nanosystem is shown in **Figure** **2**A. First, according to the traditional solvothermal method, meso‐tetrakis (4‐formylphenyl) porphyrin (P) and terephthalic dihydrazide were thoroughly mixed and heated at 120 °C to obtain P‐COF. Second, Cu was coordinated to the P‐COF backbone to acquire a metal‐ions‐decorated P‐COF complex named CuP‐COF. By stirring CuP‐COF with PEG solution, the PEGylation of CuP‐COF (PEG‐CuP‐COF) was successfully prepared. The representative transmission electron microscopy (TEM) image of PEG‐CuP‐COF was shown in Figure 2B, revealing that PEG‐CuP‐COF nanoparticles were spherical morphologies. In the elemental mapping images, Cu appeared as expected in CuP‐COF and PEG‐CuP‐COF nanoparticles, confirming the successful coordination of Cu, and other elements (C, N, O, P) remained consistent which showed that the addition of Cu and the PEGylation process did not cause significant disruption to the core structure (Figure 2C). Besides, the successful synthesis of COFs nanosystem with imine bond was illustrated by the characteristic ─C═N─ stretching vibration peaks exhibited in Fourier transform infrared (FT‐IR) spectra (Figure 2D). The X‐ray diffraction (XRD) was applied to examine the crystalline structure of COFs, and the data further showed that the crystalline structure of COFs was not destroyed after being coordinated with Cu or coated with PEG (Figure 2E). Additionally, the particle sizes of P‐COF, CuP‐COF, and PEG‐CuP‐COF were examined using the dynamic light scattering (DLS), and the average diameter was 116.20, 131.63, and 145.27 nm, respectively (Figure 2F). The zeta potential measurements revealed that P‐COF exhibited a strong positive charge of 14.75 mV. Following Cu decoration, the zeta potential of CuP‐COF significantly dropped to −10.50 mV. However, after PEGylation, it shifted back to a positive charge of 4.72 mV (Figure 2G). All these above results confirmed that the PEG‐CuP‐COF nanoparticles were successfully synthesized. Following that, the stability of PEG‐CuP‐COF was evaluated and the results showed that it had a high degree of stability not only in the neutral condition (pH7.4) but also in the acidic condition (pH6.5) (Figure S1, Supporting Information). Meanwhile, the stability of P, PEG‐P‐COF and PEG‐CuP‐COF under US irradiation was also explored using UV–vis absorption spectra (Figure 2H; Figure S2, Supporting Information). The spectra showed that the peak absorbance of P gradually decreased under US irradiation, while very little has changed in the absorbance of PEG‐P‐COF and PEG‐CuP‐COF. Thus, the COF nanosystem exhibited relatively high US stability due to the spatial constraints on porphyrin molecules within the COF structures, effectively preventing changes in molecular structure.

Next, in order to prepare the self‐driven PEG‐CuP‐COF@∆St nanosystem targeting tumor region with hypoxic conditions, PEG‐CuP‐COF nanoparticles have been innovatively anchored on the surface of ∆St through the amide condensation reaction between ‐NH_2_ on the PEG‐CuP‐COF and ‐COOH on the surface of ΔSt (Figure S3, Supporting Information). To optimize the feed ratio between PEG‐CuP‐COF and ∆St, the toxicity of PEG‐CuP‐COF to the bacteria was explored by measuring bacterial viability under different concentrations of PEG‐CuP‐COF. It was shown that there was no decrease in ∆St activity when the concentration of PEG‐CuP‐COF was 1.0 mg mL^−1^, whereas the colony‐forming units (CFU) obviously dropped when stirring with 2.0 mg mL^−1^ PEG‐CuP‐COF for 12 h (Figure S4A,B, Supporting Information). The Cell Counting Kit‐8 (CCK‐8) results also illustrated that the high concentration of PEG‐CuP‐COF had a huge negative impact on ΔSt (Figure S4C, Supporting Information). It indicated that the viability rate of ΔSt was rather low at 50.46% with a ratio of 2.0 mg PEG‐CuP‐COF/10^6^ CFU ΔSt, while the viability rate was 84.20% at the ratio of 1.0 mg PEG‐CuP‐COF/10^6^ CFU ΔSt. Thus, to ensure a high bacterial activity, PEG‐CuP‐COF@ΔSt was prepared by the ratio of 1.0 mg PEG‐CuP‐COF/10^6^ CFU ΔSt. The TEM elemental mapping images of PEG‐CuP‐COF@∆St showed that the surface of ∆St was not smooth due to the numerous linked nanoparticles and the presence of C, N, O, P, and Cu signals was further verified by the energy‐dispersive X‐ray spectroscopy (EDS) (Figure 2I,J). Moreover, the average zeta potential of PEG‐CuP‐COF@∆St was −4.69 mV, indicating an improvement from −8.02 mV observed for ∆St (Figure 2K). All these above suggested that the living ∆St bacteria was successfully linked with PEG‐CuP‐COF to acquire PEG‐CuP‐COF@∆St nanosystem.

### 2.2 Sonodynamic Performance and Multienzyme‐Mimicking Activities of PEG‐CuP‐COF

**Figure 3:**
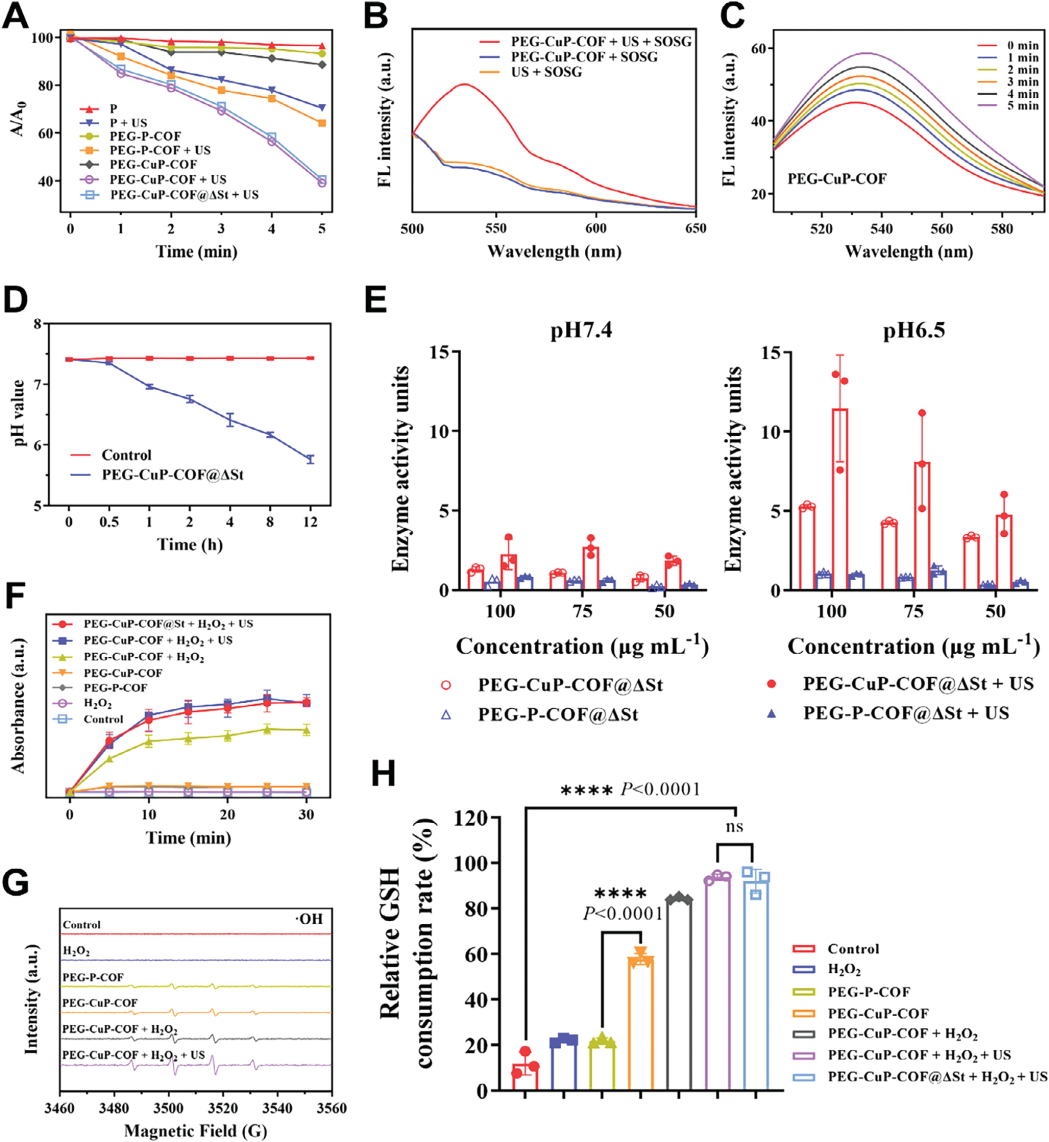
Sonodynamic performance and multienzyme‐mimicking activities of PEG‐CuP‐COF. A) UV–vis absorption degradation of DPBF under US irradiation (1.0 MHz, 1.0 W cm^−2^, 50% duty cycle). B) Fluorescence spectra of SOSG in the presence of PEG‐CuP‐COF with or without US irradiation (1.0 MHz, 1.0 W cm^−2^, 50% duty cycle, 5 min). C) DCF fluorescence spectra of PEG‐CuP‐COF under US irradiation (1.0 MHz, 1.0 W cm^−2^, 50% duty cycle). D) The pH values of medium at different time points. E) SOD‐mimicking activity of PEG‐CuP‐COF at different pH levels. F) POD‐mimicking activity of PEG‐CuP‐COF. G) Electron spin resonance (ESR) spectra of •OH trapped by DMPO. H) GPx‐mimicking activity of PEG‐CuP‐COF. Data are presented as Mean ± SD. ^****^ *P* < 0.0001, ^***^ *P* < 0.001, ^**^ *P* < 0.01, ^*^ *P* < 0.05, ns: no significance.

To explore the sonodynamic performance of PEG‐CuP‐COF nanosystem, 1,3‐Diphenylisobenzofuran (DPBF), singlet oxygen sensor green (SOSG) and 2,7‐ Dichlorodihydrofluorescein (DCFH) were used as ROS probes to evaluate the capability of ROS generation. DPBF, as a single‐linear oxygen (^1^O_2_) probe, could investigate the capability of nanosystem to yield ROS by recording the absorbance intensity at 425 nm. Generally, the more the absorbance intensity decreases, the more ROS generated. As displayed in **Figure** **3**A, the absorbance intensity of DPBF at 425 nm sharply declined with increasing US irradiation time, indicating that the PEG‐CuP‐COF and PEG‐CuP‐COF@ΔSt nanosystem could efficiently generate ROS under US irradiation. However, P, PEG‐P‐COF, and PEG‐CuP‐COF did not show ROS generation without US irradiation. The ^1^O_2_ probe SOSG was also used to confirm the ROS generation by observing the fluorescence intensity. And the results revealed that PEG‐CuP‐COF nanosystem could yield increased SOSG‐specific fluorescence signals with US irradiation (Figure 3B). Furthermore, the fluorescence intensity of DCF at 525 nm increased gradually with increasing US irradiation time, suggesting that PEG‐CuP‐COF nanosystem had excellent sonodynamic performance (Figure 3C). Notably, PEG‐P‐COF showed higher fluorescence intensity compared to P, indicating a higher level of ROS generation, because the porous structure and large surface area of COFs could enhance ROS generation by providing more active sites for energy accumulation during US irradiation (Figure S5, Supporting Information). Besides, copper ions in COFs could also catalyze ROS formation via redox reactions, significantly boosting ROS yield.

Next, the multienzyme‐mimicking activities of PEG‐CuP‐COF were systematically evaluated by investigating the substrates or products via simulating the natural enzymes in physiological conditions. The SOD‐mimicking activity of PEG‐CuP‐COF was initially explored using water‐soluble tetrazolium salt (WST), as a superoxide anion radical (O_2_^−•^) probe which could react with O_2_^−•^ to generate a kind of colored formazan spectrophotometrically monitored at 450 nm (Figure S6A, Supporting Information). Ordinarily, O_2_^−•^ was generated from the xanthine with Xanthine Oxidase (XO) catalyst, whereas SOD mimics could quench O_2_^−•^ to yield H_2_O_2_ and O_2_. Besides, considering the decreased pH value in the cultivation environment caused by ΔSt breaking sugar molecules down into various acids, the SOD‐mimicking activity at different pH levels (7.4 and 6.5) was investigated, respectively (Figure 3D,E). It was shown that both nanosystems showed relatively low enzyme‐mimicking activity at pH7.4, with no significant increase as the concentration rises. At pH6.5, the SOD‐mimicking activity of PEG‐CuP‐COF@ΔSt significantly increased, especially at higher concentrations and under US irradiation, while the activity of PEG‐P‐COF@ΔSt remained largely unchanged even with high concentration or under US irradiation. These results suggested that the PEG‐CuP‐COF possessed excellent SOD‐mimicking activity even in the PEG‐CuP‐COF@∆St formulation and demonstrated much higher SOD‐mimicking activity compared with PEG‐P‐COF in more acidic conditions, possibly due to more favorable redox reactions of copper ions at lower pH. Specifically, the copper ions in COF structures played a key role in mimicking SOD activity and the redox potential of copper ions could shift to more favorable values at lower pH, thus enhancing the electron transfer required for the catalytic cycle.^[^ ^26^ ^]^ Moreover, the dismutation reaction of superoxide involved both reduction and oxidation processes, where protons (H⁺) were consumed in the conversion of superoxide to hydrogen peroxide (H_2_O_2_).^[^ ^25^ ^]^ At lower pH, the increased proton availability could facilitate this reaction, accelerating the catalytic cycle of SOD‐mimicking activity.

The POD‐mimicking activity of PEG‐CuP‐COF by catalyzing H_2_O_2_ to hydroxyl radicals (•OH) was measured using 3,3′,5,5′‐ tetramethylbenzidine (TMB), as an •OH probe which could be oxidized by •OH into blue‐colored oxidized TMB (oxTMB) with a maximum absorption peak at 654 nm. As indicated in Figure 3F, there was a significant time‐dependent enhancement in the absorption at 654 nm when put PEG‐CuP‐COF or PEG‐CuP‐COF@ΔSt to the aqueous solution containing H_2_O_2_ and US irradiation, suggesting that PEG‐CuP‐COF exhibited outstanding POD‐mimicking activity even in the PEG‐CuP‐COF@∆St formulation. Furthermore, the solution without PEG‐CuP‐COF or H_2_O_2_ demonstrated negligible color variation and had no increase in the absorption peak (Figure S6B, Supporting Information). In addition, •OH yielded from H_2_O_2_ with PEG‐CuP‐COF catalyst was further measured by electron spin resonance (ESR) spectra using 5,5‐dimethyl‐1‐pyrroline‐N‐oxide (DMPO) as the trapping agent (Figure 3G). The PEG‐CuP‐COF with H_2_O_2_ addition displayed characteristic •OH signals, further confirming the POD‐mimicking activity of PEG‐CuP‐COF. Notably, the sharpest signals of •OH appeared in the mixture containing PEG‐CuP‐COF and H_2_O_2_ under US irradiation, probably because US could promote enzyme‐catalyzed reactions by reducing the mass diffusion barrier in nanocatalysts.^[^ ^40^, ^41^ ^]^

To detect the GPx‐mimicking activity of PEG‐CuP‐COF, 5,5′‐dithiobis (2‐nitrobenzoic acid) (DTNB) was used as glutathione (GSH) probe which could react with GSH to yield yellow‐colored water‐soluble product spectrophotometrically monitored at 412 nm. The yellow color of the aqueous solution would gradually lighten as GP_X_ mimics catalyzed the reduction of GSH to oxidized glutathione (GSSG) accompanying by the conversion of reduced H_2_O_2_ to H_2_O (Figure S6C, Supporting Information). As shown in Figure 3H, PEG‐CuP‐COF and PEG‐CuP‐COF@∆St exhibited excellent GPx‐mimicking activity, catalyzing nearly 80% of the GSH consumption. Moreover, the GPx‐mimicking activity of PEG‐CuP‐COF could be enhanced to a certain extent under US irradiation.

Summarily, the sonodynamic performance and multienzyme‐mimicking activities of PEG‐CuP‐COF formed a virtuous circle of promotion. Briefly, the generated ROS improved the multienzyme‐catalyzed reactions by acting as free radicals, participating in reactions and generating intermediates that further facilitated the reaction, and in turn the multienzyme‐catalyzed reactions enhanced sonodynamic efficiency through the catalytically produced •OH and consumption of GSH. To validate the auxiliary role of multienzyme‐catalyzed reactions in enhancing sonodynamic effects, we assessed the viability of RM‐1 cells treated with different conditions using the standard Cell Counting Kit‐8 (CCK‐8) assay (Figure S7, Supporting Information). The results indicated that the PEG‐CuP‐COF + H_2_O_2_ group showed lower cell viability than the PEG‐P‐COF + H_2_O_2_ group, because of the production of •OH and consumption of GSH. Moreover, compared with the PEG‐CuP‐COF + H_2_O_2_, PEG‐CuP‐COF + US, and PEG‐P‐COF + H_2_O_2_ + US groups, the cell viability in the PEG‐CuP‐COF + H_2_O_2_ + US group was significantly reduced, suggesting that the PEG‐CuP‐COF with a combination of SDT and multienzyme‐catalyzed reactions would achieve better antitumor effects. Overall, the catalytically produced •OH improved the cytotoxicity of sonodynamic effect, and the sonodynamic efficacy could be enhanced simultaneously due to the impaired antioxidant defense resulting from GSH depletion.

### 2.3 Cellular Uptake and Cytotoxicity of PEG‐CuP‐COF

**Figure 4:**
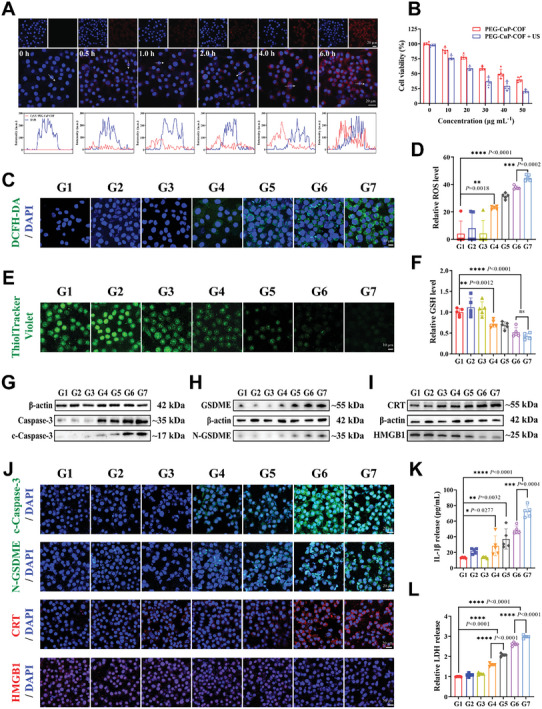
ROS‐activated pyroptosis and therapeutic efficacy of PEG‐CuP‐COF in vitro. A) Intracellular co‐localization of Cy5.5‐labeled PEG‐CuP‐COF in RM‐1 cells after incubation for different time intervals (0, 0.5, 1, 2, 4, and 6 h). B) Cell viability of RM‐1 cells after incubation with different concentrations of PEG‐CuP‐COF with or without US irradiation. C) Representative fluorescence images of DCFH‐DA staining of RM‐1 cells and D) corresponding fluorescence intensity for different groups. E) Representative fluorescence images of ThiolTracker Violet staining of RM‐1 cells and F) corresponding fluorescence intensity for different groups. G,H) Western blot analysis of expression levels of pyroptosis‐related proteins (Caspase‐3, c‐Caspase‐3, GSDME, and N‐GSDME) in RM‐1 cells for various groups. I) Western blot analysis of expression levels of DAMPs‐related proteins (CRT and HMGB1) in RM‐1 cells for various groups. J) Representative immunofluorescence images of c‐Caspase‐3, N‐GSDME, CRT, and HMGB1 of RM‐1 cells for various groups. K) IL‐1β release from RM‐1 cells after different treatments. L) LDH release from RM‐1 cells after different treatments. G1: Control, G2: H_2_O_2_, G3: US only, G4: PEG‐CuP‐COF, G5: PEG‐CuP‐COF + H_2_O_2_, G6: PEG‐CuP‐COF + US, G7: PEG‐CuP‐COF + H_2_O_2_ + US. Data are presented as Mean ± SD. ^****^ *P* < 0.0001, ^***^ *P* < 0.001, ^**^ *P* < 0.01, ^*^ *P* <0.05, ns: no significance.

After investigating the multienzyme‐mimicking activities and sonodynamic performance, the internalization and trafficking processes of PEG‐CuP‐COF nanoparticles were initially explored by observing the location of PEG‐CuP‐COF in RM‐1 cells through the confocal laser scanning microscopy (CLSM). Firstly, the cellular uptake of PEG‐CuP‐COF nanoparticles was visualized at different time points after co‐incubation with RM‐1 cells and the co‐localization of Cy5.5‐labeled red fluorescence of PEG‐CuP‐COF nanoparticles with blue fluorescence of cell nucleus was also analyzed (**Figure** **4**A). As displayed in CLSM images, the phagocytosis of PEG‐CuP‐COF nanoparticles started at 0.5 h, saturated at 4 h and lasted until 6 h after co‐incubation with RM‐1 cells. Subsequently, the location of PEG‐CuP‐COF nanoparticles in organelles with or without US irradiation was further visualized. Under US irradiation, the Cy5.5‐labeled red fluorescence of PEG‐CuP‐COF nanoparticles was dissociated with the green fluorescence of lysosomes, suggesting that US irradiation could promote enhanced lysosomal escape (Figure S8, Supporting Information). These results indicated that PEG‐CuP‐COF nanoparticles could achieve major cellular uptake and efficient retention in tumor cells, which was a prerequisite for PEG‐CuP‐COF nanoparticles to trigger pyroptosis in the cytoplasm.

Next, the cytotoxicity of PEG‐CuP‐COF nanosystem was evaluated by standard CCK‐8 assay (Figure S9, Supporting Information). The Alpha mouse liver 12 (AML‐12) cells and normal mouse prostate epithelial cells (PrECs) were respectively treated with various concentrations of PEG‐CuP‐COF for 8 h and more than 85% of cell viability was reached after co‐incubation with 30 µg mL^−1^ PEG‐CuP‐COF. Notably, the PEG‐CuP‐COF exhibited greater cytotoxicity to RM‐1 cells primarily because of the higher H_2_O_2_ levels in tumor cells. And the PEG‐CuP‐COF achieved a higher mortality rate of tumor cells under US irradiation due to the increased ROS generation, indicating the essential of US irradiation in antitumor therapy (Figure 4B).

### 2.4 ROS‐Activated Pyroptosis and Therapeutic Efficacy of PEG‐CuP‐COF In Vitro

To investigate the ROS‐activated pyroptosis and therapeutic efficacy of PEG‐CuP‐COF in vitro, RM‐1 tumor cells were employed and given different treatments including G1: Control, G2: H_2_O_2_, G3: US only, G4: PEG‐CuP‐COF, G5: PEG‐CuP‐COF + H_2_O_2_, G6: PEG‐CuP‐COF + US, and G7: PEG‐CuP‐COF + H_2_O_2_ + US. Initially, the intracellular ROS generation in various groups was explored by DCFH‐DA staining. The images displayed that negligible fluorescence was detected in RM‐1 cells subjected to H_2_O_2_, US or PEG‐CuP‐COF alone, while the PEG‐CuP‐COF + US and PEG‐CuP‐COF + H_2_O_2_ + US groups generated a large amount of ROS, confirming that PEG‐CuP‐COF nanosystem could yield abundant ROS with US irradiation to further promote pyroptosis‐related cell death (Figure 4C,D). The GPx‐mimicking activity of PEG‐CuP‐COF nanosystem was also evaluated at cellular level by comparing GSH contents in different groups. In the tumor cells treated with PEG‐CuP‐COF, the green fluorescence intensity of GSH was obviously weakened in comparison to the control group (Figure 4E,F). Significantly, inappreciable fluorescence signal was observed with extra US irradiation, revealing that PEG‐CuP‐COF nanosystem exerted a strong GPx‐mimicking activity to lower cellular GSH level especially under US irradiation. Considering that the presence of high‐level GSH may defend tumor cells against ROS attack through powerful antioxidant systems, thus PEG‐CuP‐COF nanosystem could further enhance antitumor effects via GSH depletion.

In addition, the antitumor efficacy of PEG‐CuP‐COF nanosystem in vitro was explored by Calcein‐AM/PI staining (Figure S10, Supporting Information). Consistent with CCK‐8 results, the PEG‐CuP‐COF + US and PEG‐CuP‐COF + H_2_O_2_ + US groups displayed a larger amount of PI‐stained dead tumor cells with red fluorescence than other groups, indicating the outstanding performance in killing tumor cells. The flow cytometry (FCM) was further conducted to investigate cell apoptosis and the results revealed that the PEG‐CuP‐COF + H_2_O_2_ + US group showed a significant increase in the apoptosis ratio as expected (Figure S11, Supporting Information).

Subsequently, to explore the mechanism of ROS‐activated pyroptosis, the proteins associated with pyroptosis were detected by Western blot. From the Western blot analysis, the band intensities of N‐terminal fragments of GSDME (N‐GSDME) and cleaved Caspase‐3 (c‐Caspase‐3) in RM‐1 cells were sharply enhanced after co‐incubation with PEG‐CuP‐COF, and their expressions could be further promoted with US irradiation (Figure 4G,H; Figure S12A, Supporting Information). Notably, despite the full‐length GSDMD detected in all the groups, there was no obvious expressions of N‐terminal fragments of GSDMD (N‐GSDMD) (Figure S13, Supporting Information). These results revealed that PEG‐CuP‐COF nanosystem could trigger pyroptosis along with Caspase‐3 activation and subsequent GSDME cleavage, which was significantly different to the canonical GSDMD‐dependent pyroptosis. Additionally, the damage‐associated molecular patterns (DAMPs)‐related proteins including calreticulin (CRT) and high‐mobility group box 1 protein (HMGB1) were also detected by Western blot. It was shown that the up‐regulation of CRT and down‐regulation of HMGB1 appeared mainly in the PEG‐CuP‐COF + US and PEG‐CuP‐COF + H_2_O_2_ + US groups, indicating the efficient immunogenic cell death of pyroptosis caused by PEG‐CuP‐COF and US (Figure 4I; Figure S12B, Supporting Information). These findings were further confirmed by immunofluorescence staining, and the pyroptosis‐related proteins and DAMPs displayed a similar expression trend (Figure 4J; Figure S14, Supporting Information). Finally, as depicted in Figure 4K,L, the release of intracellular contents such as interleukin‐1β (IL‐1β) and lactate dehydrogenase (LDH) which were commonly regarded as typical pro‐inflammatory cytokines about cell death, significantly increased in the PEG‐CuP‐COF + US and PEG‐CuP‐COF + H_2_O_2_ + US groups. Taken together, all of the results proved that the large amount of ROS generated by PEG‐CuP‐COF under US irradiation could efficiently activate GSDME‐dependent pyroptosis, accompanying by the release of abundant DAMPs and pro‐inflammatory cytokines.

### 2.5 Biosafety of PEG‐CuP‐COF@∆St Nanosystem In Vivo

Initially, the safe therapeutic dose of ∆St bacteria was determined by recording the body weight change in healthy mice during 30 days after injection of different doses of bacteria. It was shown that the excessive dose of ΔSt (200 µL per mouse, 10^7^ CFU mL^−1^) led to an early weight loss, while the lower dose (200 µL per mouse, 10^6^ CFU mL^−1^) was more secure and feasible (Figure S15, Supporting Information). Therefore, the dose of 10^6^ CFU mL^−1^ was used for later experiments in vivo. Subsequently, the biosafety of PEG‐CuP‐COF@∆St nanosystem was further evaluated by hematoxylin and eosin (H&E) staining of major organs (heart, liver, spleen, lung, and kidney) and blood biochemistry. The major organs of mice were harvested for H&E staining at various time intervals (1, 3, 7, and 30 days) after PEG‐CuP‐COF@∆St administration (200 µL per mouse, 1 mL PEG‐CuP‐COF@ΔSt contained 1 × 10^6^ CFU ΔSt and 1.0 mg PEG‐CuP‐COF), and the results demonstrated that no obvious necrosis or inflammation was observed (Figure S16A, Supporting Information). Then the blood was collected for hematological examination, wherein no significant difference in the biochemical indexes such as blood, liver, and kidney functions existed in the experimental mice (Figure S16B,C, Supporting Information). Additionally, LB agar plate counts were conducted for major organs, which displayed that the heart, lung, and kidney were free of ∆St whereas minor residues were in the liver and spleen after tail vein injection of PEG‐CuP‐COF@∆St nanosystem for 30 days (Figure S17, Supporting Information).

### 2.6 Tumor‐Targeting Characterization of PEG‐CuP‐COF@∆St

**Figure 5:**
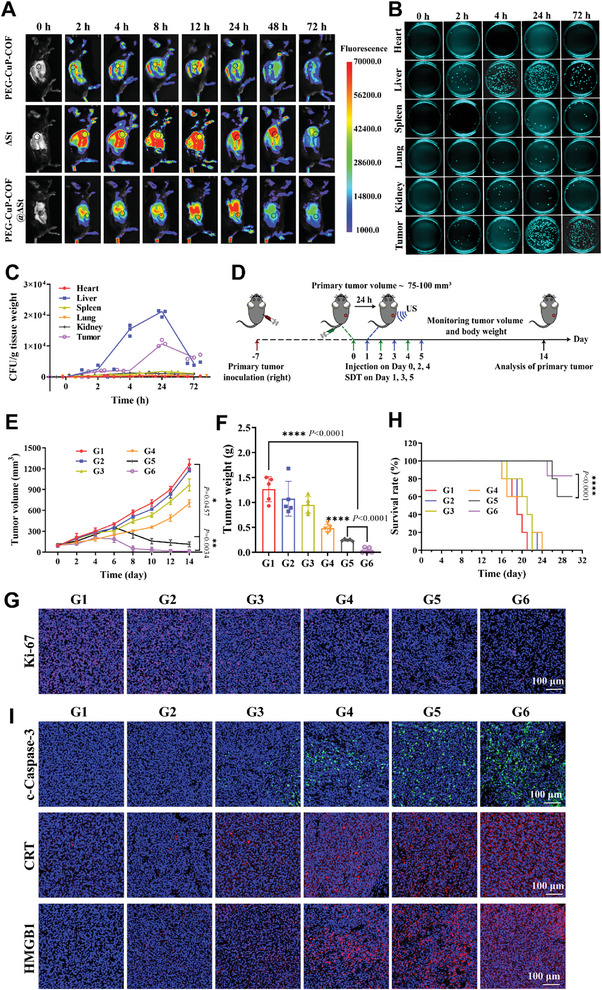
Effectiveness of PEG‐CuP‐COF@∆St nanosystem against primary tumors. A) In vivo fluorescence images of Cy5.5‐labeled PEG‐CuP‐COF, Cy5.5‐labeled ∆St, and Cy5.5‐labeled PEG‐CuP‐COF@∆St in mice at different time points. B) Typical images of LB agar plates and C) corresponding CFU quantification of bacterial colonization in various organs after injection of PEG‐CuP‐COF@∆St. D) Schematic illustration of primary tumor model treatment. E) Average tumor growth curves in different groups. F) Average weight of excised tumor tissues in each group. G) Representative images of Ki‐67 immunofluorescence staining in tumor sections from various groups. H) Survival curves of mice with different treatments (*n* = 5). I) Representative images of c‐Caspase‐3, CRT, and HMGB1 immunofluorescence staining in tumor sections from various groups. G1: Control, G2: US only, G3: PEG‐CuP‐COF, G4: PEG‐CuP‐COF@∆St, G5: PEG‐CuP‐COF + US, G6: PEG‐CuP‐COF@∆St + US. Data are presented as Mean ± SD. ^****^ *P* < 0.0001, ^***^ *P* < 0.001, ^**^ *P* < 0.01, ^*^ *P* < 0.05, ns: no significance.

To achieve nano delivery and antitumor therapy in vivo, the tumor‐targeting capability of PEG‐CuP‐COF@∆St nanosystem was deeply explored in RM‐1 tumor‐bearing mice. The Cy5.5‐labeled PEG‐CuP‐COF, Cy5.5‐labeled ∆St, and Cy5.5‐labeled PEG‐CuP‐COF@∆St were respectively administered to RM‐1 tumor‐bearing mice, and fluorescence images in vivo were acquired at different time points after intravenous injection. The fluorescence images displayed that the Cy5.5 intensity at tumor site increased over time and reached the highest at 24 h, and was stronger in the PEG‐CuP‐COF@∆St group compared to the PEG‐CuP‐COF group, suggesting that PEG‐CuP‐COF@∆St nanosystem had a better tumor‐enriching property (**Figure** **5**A; Figure S18, Supporting Information). To investigate ∆St proliferation in tumor tissues and major organs, the tissues were collected and homogenized for spreading on LB agar plates at 24 h after injection. The results revealed that no bacterial was observed in the PEG‐CuP‐COF group, while markedly proliferating in the livers and tumors in ∆St and PEG‐CuP‐COF@∆St groups (Figure S19, Supporting Information). Additionally, LB agar plate counts were performed at different time points for tumor tissues and major organs in the PEG‐CuP‐COF@∆St‐administrated group, which displayed that ∆St gradually removed from heart, liver, spleen, lung and kidney whereas increased significantly over time in liver and tumor tissues within 24 h, suggesting the excellent tumor‐targeting property of PEG‐CuP‐COF@∆St nanosystem (Figure 5B,C).

Moreover, the major organs and tumor tissues were also harvested for *ex vivo* fluorescence imaging and corresponding fluorescence intensity analysis at 8, 24, and 72 h after injection (Figure S20, Supporting Information). The results suggested that the ∆St and PEG‐CuP‐COF@∆St groups accumulated stronger signals in tumors compared with the PEG‐CuP‐COF group, which was attributed to the hypoxic microenvironment in tumors eminently suitable for ∆St proliferation. Generally, the self‐driven PEG‐CuP‐COF@∆St nanosystem exhibited favorable tumor‐targeting characteristic, and could efficiently deliver PEG‐CuP‐COF to accumulate in tumor sites.

### 2.7 Effectiveness of PEG‐CuP‐COF@∆St Nanosystem in the Treatment of Primary Tumors In Vivo

Motivated by the great therapeutic efficacy in vitro and excellent tumor‐targeting characterization, the antitumor effects of PEG‐CuP‐COF@∆St nanosystem were investigated in vivo. As displayed in Figure 5D, RM‐1 cells were subcutaneously implanted into the right flank of C57BL/6 mice to establish tumor models, and after tumor growth for 7 days the tumor‐bearing mice were randomly divided into six groups including G1: Control, G2: US only, G3: PEG‐CuP‐COF, G4: PEG‐CuP‐COF@∆St, G5: PEG‐CuP‐COF + US and G6: PEG‐CuP‐COF@∆St + US. The PEG‐CuP‐COF or PEG‐CuP‐COF@ΔSt was intravenously injected to the designated groups on days 0, 2 and 4, followed by US irradiation at day one post‐injection. The tumor volume and body weight were regularly recorded every two days until day 14. The final results showed that the tumor growth in G3 was slightly delayed compared to that in G1 and G2, indicating that PEG‐CuP‐COF alone possessed a mild effect in inhibiting tumor progression (Figure 5E; Figure S21A, Supporting Information). And the inhibitory effect of G4 was more evident than that of G3, which was originated from the proven tumor‐targeting property of ΔSt. Notably, due to the efficient SDT performance of PEG‐CuP‐COF, the tumor growth in G5 and G6 was significantly slower than that in G3 and G4. Furthermore, the excised‐tumor image and tumor weight in various groups were also acquired, and the results indicated that the tumors in G6 could be mostly eliminated after a treatment period, demonstrating an excellent antitumor efficacy of PEG‐CuP‐COF@ΔSt nanosystem‐mediated pyroptosis (Figure 5F; Figure S21B, Supporting Information). The pathological changes in tumor tissues were detected through deoxynucleotidyl transferase‐mediated dUTP‐biotin nick end labeling (TUNEL) and H&E staining, and the images displayed a great number of necrotic cells in G5 and G6, which was more obvious in G6 (Figure S21C–E, Supporting Information). This result demonstrated that the strategy of PEG‐CuP‐COF@ΔSt + US definitely possessed a favorable therapeutic efficacy in the treatment of primary tumors. To further confirm the antitumor effect in G6, Ki‐67 staining was performed on tumor tissues. And the staining image in G6 showed a markedly decreased expression of Ki‐67 compared with other groups, suggesting the significant inhibitory proliferation effect of tumor cells (Figure 5G; Figure S21F, Supporting Information). Moreover, the survival curve revealed that the treatment strategy of PEG‐CuP‐COF@ΔSt + US could prolong mice survival with no significant change in body weight, which further confirmed the outstanding biosafety of PEG‐CuP‐COF@ΔSt (Figure 5H; Figure S21G, Supporting Information).

The tumor pyroptosis induced by PEG‐CuP‐COF@ΔSt in vivo was explored through the detection of related biomarkers. Initially, the expression level of c‐Caspase‐3 in tumor tissues was observed by immunofluorescence staining (Figure 5I; Figure S21H, Supporting Information). The results showed that the c‐Caspase‐3 level was obviously up‐regulated in the PEG‐CuP‐COF@ΔSt + US group, which revealed the occurrence of pyroptosis during cell death. And it also confirmed that the designed nanosystem could improve the targeting delivery of PEG‐CuP‐COF and further mediate pyroptosis to enhance antitumor effects. Subsequently, the immunofluorescence staining for DAMPs‐related proteins (CRT and HMGB1) was administrated to explore the antitumor responses induced by pyroptosis (Figure 5I; Figure S21I,J, Supporting Information). The findings showed that CRT and HMGB1 were strongly expressed in the PEG‐CuP‐COF@ΔSt + US group, demonstrating that PEG‐CuP‐COF@ΔSt nanosystem with US irradiation could promote the immunogenicity of tumor cells. Collectively, it was concluded that PEG‐CuP‐COF@ΔSt nanosystem combined with US irradiation could efficiently trigger immunogenic pyroptosis to achieve a powerful antitumor effect in vivo.

### 2.8 Antitumor Immune Responses In Vivo

**Figure 6:**
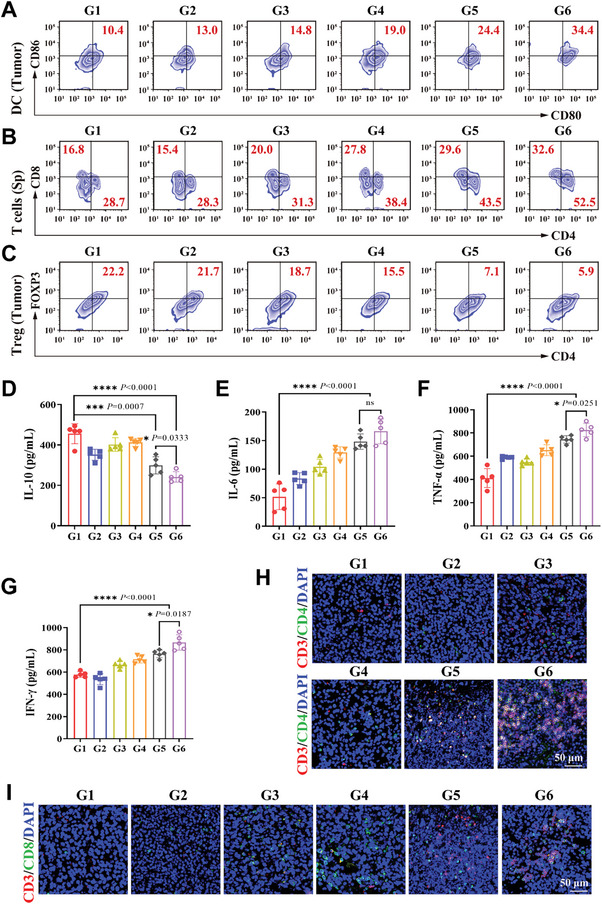
In vivo analysis of immune effects. A) Typical flow cytometry of mature dendritic cells (CD11c^+^CD80^+^CD86^+^, DCs) in primary tumors after initial various treatments. B) Typical flow cytometry of CD4^+^ and CD8^+^ T cells in the spleen (Sp) after initial various treatments. C) Typical flow cytometry of regulatory T cells (CD3^+^CD4^+^Foxp3^+^, Tregs) in primary tumor tissues after initial various treatments. D–G) Intra‐tumor levels of IL‐10, IL‐6, TNF‐α, and IFN‐γ. H,I) Immunofluorescence images of tumor tissues infiltrating CD3^+^CD4^+^ and CD3^+^CD8^+^ T cells after initial various treatments. G1: Control, G2: US, G3: PEG‐CuP‐COF, G4: PEG‐CuP‐COF@∆St, G5: PEG‐CuP‐COF + US, G6: PEG‐CuP‐COF@∆St + US. Data are presented as Mean ± SD. ^****^ *P* < 0.0001, ^***^ *P* < 0.001, ^**^ *P* < 0.01, ^*^ *P* < 0.05, ns: no significance.

To explore the antitumor immune responses initiated by PEG‐CuP‐COF nanosystem‐mediated pyroptosis, the immune cells in various groups were detected by flow cytometry. The mature dendritic cells (CD11c^+^CD80^+^CD86^+^, DCs) in tumor tissues was firstly examined. It was displayed that the proportion of mature DCs was significantly increased with administration of PEG‐CuP‐COF, and reached the highest average of 35.10% in the PEG‐CuP‐COF@∆St + US group, which demonstrated that the PEG‐CuP‐COF nanosystem‐mediated pyroptosis could effectively induce DCs maturation (**Figure** **6**A; Figure S22A, Supporting Information). Besides, the average proportions of mature DCs in the spleen were ≈31.68% in the PEG‐CuP‐COF + US group and 43.62% in the PEG‐CuP‐COF@∆St + US group, which were higher than that in groups without US, revealing that DCs maturation could be promoted under US irradiation (Figure S22B,C, Supporting Information). Meanwhile, the flow cytometry results showed an increase in the proportion of CD4^+^ and CD8^+^ T cells after treatment with PEG‐CuP‐COF@∆St + US, and confirmed that the PEG‐CuP‐COF@ΔSt nanosystem‐mediated pyroptosis could also facilitate lymphocyte responses (Figure 6B; Figure S22D–F, Supporting Information). Notably, an obvious decrease in the proportion of regulatory T cells (CD3^+^CD4^+^Foxp3^+^, Tregs) was also seen in the PEG‐CuP‐COF@∆St + US group, demonstrating that PEG‐CuP‐COF@∆St + US‐mediated pyroptosis effectively reduced immunosuppression and remodeled tumor microenvironment (Figure 6C; Figure S22G, Supporting Information).

The status of activated immune system was further explored by examining the intra‐tumor levels of cytokines secreted by immune cells. As shown in Figure 6D–F, inflammatory factors were detected to investigate the effects of immune activation with various treatments. Compared with other groups, the anti‐inflammatory factor interleukin‐10 (IL‐10) was produced at a lower level in the PEG‐CuP‐COF@∆St + US group, while the interleukin‐6 (IL‐6) and tumor necrosis factor‐α (TNF‐α), two kinds of typical pro‐inflammatory factors, were obviously up‐regulated. Moreover, interferon‐γ (IFN‐γ) was effectively produced under the treatment of PEG‐CuP‐COF@∆St + US, confirming the activation of T cells and the enhanced immune system activity (Figure 6G). The activation of T cells could be also proved by immunofluorescence staining of Granzyme B (GzmB) which was closely related to cytotoxic T cell‐mediated antitumor treatment, and an obvious overexpression was displayed in the PEG‐CuP‐COF@∆St + US group (Figure S22H,I, Supporting Information).

Subsequently, the immunofluorescence staining of tumor‐infiltrating helper T lymphocytes (HTLs, CD3^+^CD4^+^) and cytotoxic T lymphocytes (CTLs, CD3^+^CD8^+^) was performed to further demonstrate the antitumor immune response (Figure 6H,I; Figure S22J,K, Supporting Information). As a result, the proliferation of HTLs and CTLs was relatively promoted in the PEG‐CuP‐COF + US and PEG‐CuP‐COF@∆St + US groups, which was more evident in the PEG‐CuP‐COF@∆St + US group, indicating that the antitumor immune system could be significantly enhanced by using the strategy of PEG‐CuP‐COF@∆St delivery nanosystem added with US irradiation. Together, these results confirmed that PEG‐CuP‐COF@∆St + US‐mediated pyroptosis could effectively stimulate T cell proliferation and activate immune responses to exert an excellent antitumor effect.

### 2.9 RNA‐Sequencing Analysis

**Figure 7:**
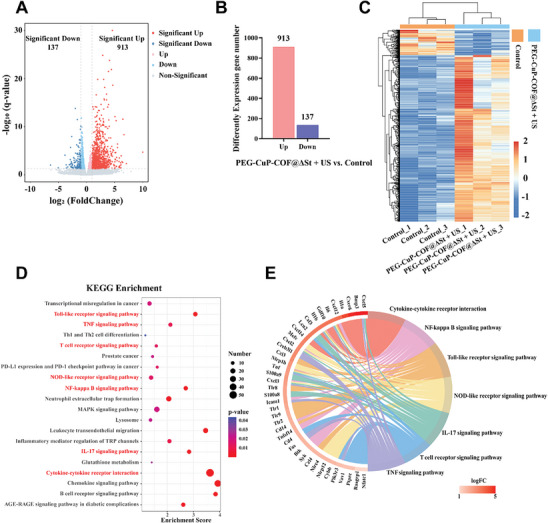
RNA‐sequencing analysis of tumor tissues after different treatments. A) Volcano plots and B) numbers of gene alteration in the PEG‐CuP‐COF@ΔSt + US group compared to the control group. C) Cluster diagram of differentially expressed genes (DEGs) between the PEG‐CuP‐COF@ΔSt + US group and the control group (*n* = 3 per group). D) KEGG enrichment analysis for studying underlying pathways after PEG‐CuP‐COF@ΔSt + US treatments. E) Circle diagram of KEGG enrichment analysis of inflammation/immune‐related DEGs.

To further investigate the underlying bio‐mechanism of PEG‐CuP‐COF@∆St in promoting therapeutic efficacy, RNA‐sequencing was conducted on tumor tissues to study the gene changes after treatments. First, six RM‐1 tumor‐bearing mice with tumor volumes ranging from 75 to 100 mm^3^ were randomly divided into the control group (without any treatment) and the treated group (PEG‐CuP‐COF@ΔSt + US). Then the treated group was given three times of intravenous injection followed by US irradiation as mentioned above. Subsequently, tumor tissues in two groups were harvested for RNA‐sequencing. The gene expression levels in the same group displayed obvious clustering indicating high reliability of the RNA‐sequencing data (Figure S23, Supporting Information). Besides, RNA‐sequencing analysis displayed 1050 differentially expressed genes (DEGs) between the treated group and the control group, in which 913 (86.95%) were up‐regulated genes and 137 (13.05%) were down‐regulated genes (**Figure** **7**A–C). Furthermore, the Kyoto Encyclopedia of Genes and Genomes (KEGG) analysis were carried out on the 913 up‐regulated DEGs (Figure 7D,E; Figure S24, Supporting Information). The results showed that these up‐regulated DEGs were enriched in multiple signaling pathways mainly including TNF (tumor necrosis factor), NF‐κB (nuclear factor‐κB), IL‐17, Toll‐like receptor (TLR), and Nod‐like receptor (NLR) signaling pathways, confirming the close correlation between PEG‐CuP‐COF@ΔSt treatment and the inflammation response. Importantly, the cytokine‐cytokine receptor interaction pathway and T cell receptor signaling pathway were also significantly enriched which suggested the potential relationship between PEG‐CuP‐COF@ΔSt treatment and the activated immune system. Additionally, the gene ontology (GO) enrichment analysis further indicated the relationship between up‐regulated DEGs and inflammation/immune‐related pathways (Figures S25 and S26, Supporting Information). It is proven that pyroptosis starts from the activation of cytosolic pattern recognition receptors including TLRs and NOD1/2, and then causes the release of pro‐inflammatory cytokines such as IL‐1β and TNF which can subsequently potentiate a strong immune response^[^ ^1^, ^2^ ^]^. In conclusion, the transcriptional changes in tumor genes caused by PEG‐CuP‐COF@ΔSt treatment were explored by RNA‐sequencing, which confirmed the essential role for PEG‐CuP‐COF@ΔSt serving as pyroptosis inducers in antitumor therapy.

### 2.10 Effectiveness of PEG‐CuP‐COF@∆St Nanosystem in Inhibiting Distant Tumors and Bone Metastases

**Figure 8:**
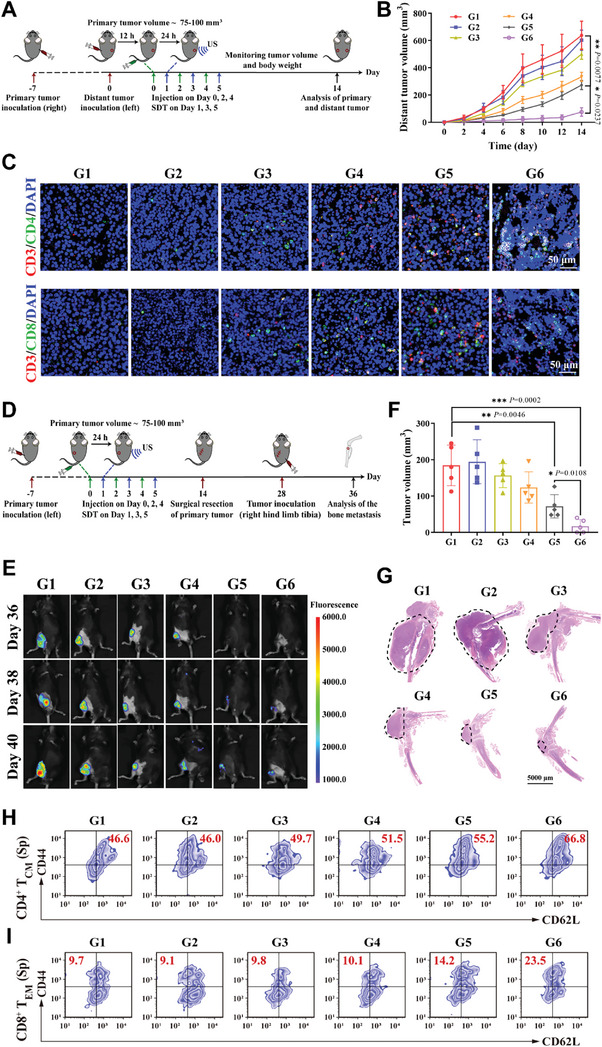
Effectiveness of PEG‐CuP‐COF@∆St nanosystem in inhibiting distant tumors and bone metastases. A) Schematic illustration of the establishment and treatment process of mice model with distant tumors. B) Average tumor growth curves in different groups. C) Immunofluorescence images of distant tumors infiltrating CD3^+^CD4^+^ and CD3^+^CD8^+^ T cells. D) Schematic illustration of the establishment of bone metastasis models. E) In vivo bioluminescence images displaying bone metastases in various groups. F) Bone tumor volumes in various groups. G) Representative H&E‐stained bone tissues in various groups. H) Typical flow cytometry of central memory T cells (CD3^+^CD4^+^CD44^+^CD62L^+^, T_CM_) in the spleen (Sp). I) Typical flow cytometry of effector memory T cells (CD3^+^CD8^+^CD44^+^CD62L^−^, T_EM_) in the spleen (Sp). G1: Control, G2: US, G3: PEG‐CuP‐COF, G4: PEG‐CuP‐COF@∆St, G5: PEG‐CuP‐COF + US, G6: PEG‐CuP‐COF@∆St + US. Data are presented as Mean ± SD. ^****^ *P* < 0.0001, ^***^ *P* < 0.001, ^**^ *P* < 0.01, ^*^ *P* < 0.05, ns: no significance.

The antitumor effect of PEG‐CuP‐COF@∆St + US therapeutic strategy was further investigated in bilateral RM‐1 tumor‐bearing mice. As depicted in **Figure** **8**A, primary tumors were firstly established by subcutaneously injecting RM‐1 cells into the right flank of C57BL/6 mice, and the distant tumors were subsequently grafted onto the left flank of mice after primary tumor growth for 7 days. Notably, US irradiation was only administrated for primary tumors, followed by bi‐daily monitoring of primary and distant tumor growth. Consistent with the results in unilateral tumor‐bearing mice, the primary tumor volume was significantly decreased in the PEG‐CuP‐COF + US and PEG‐CuP‐COF@∆St + US groups compared to other groups (Figure S27A,B, Supporting Information). Besides, the growth curves of distant tumors displayed an impressive suppression in mice treated with PEG‐CuP‐COF@∆St + US, which confirmed that this treatment strategy could also exhibit a favorable inhibitory effect on distant tumors with no significant change in the body weight (Figure 8B; Figure S28A,B, Supporting Information). Additionally, the bilateral tumor tissues were harvested and weighed after treatment ended, and the results indicated an excellent antitumor efficacy of PEG‐CuP‐COF@∆St + US both in the primary tumors and in the distant tumors (Figures S27C,D and S28C,D, Supporting Information). Further exploration into the mechanism of inhibitory effects was conducted through the immunofluorescence staining of CD3^+^CD4^+^ and CD3^+^CD8^+^ T cells infiltrating in distant tumor tissues (Figure 8C; Figure S28E,F, Supporting Information). And there was an increase in the CD3^+^CD4^+^ and CD3^+^CD8^+^ T cells in PEG‐CuP‐COF@∆St + US group, suggesting that the antitumor mechanism could be attributed to the immune system comprehensively activated by the PEG‐CuP‐COF@∆St + US‐mediated pyroptosis.

Notably, ≈90% of men with advanced prostate cancer will develop bone metastasis, which is a huge challenge facing clinical treatment due to a low survival period.^[^ ^42^ ^]^ Therefore, mice model of bone metastases was established to investigate the anti‐bone metastases effect produced by PEG‐CuP‐COF@∆St + US (Figure 8D). First, RM‐1 tumor‐bearing mice were randomly divided into six groups and subjected to different treatments as mentioned before. Then the primary tumors were removed at the end of treatment. Two weeks later, RM‐1 cells labeled with luciferase were injected into the bone marrow cavity along the long axis of the tibia for each mouse, and bioluminescence signals in bone tumors were increasingly detected. The results showed that the mice treated with PEG‐CuP‐COF + US and PEG‐CuP‐COF@∆St + US displayed an extremely weak bioluminescence signal in metastatic bone tumors, confirming that the bone metastasis could be effectively suppressed by PEG‐CuP‐COF nanosystem combined with US irradiation (Figure 8E). The metastatic bone tumors in various groups were also harvested and subjected to H&E staining, and the results indicated that the volume of metastatic bone tumors in the PEG‐CuP‐COF@∆St + US treated mice was obviously smaller than those in other groups (Figure 8F,G; Figure S29, Supporting Information). Furthermore, to explore whether PEG‐CuP‐COF@∆St + US could enhance the immune memory effect of T cells, the flow cytometry was used to measure the proportion of central and effector memory T cells (CD3^+^CD4^+^CD44^+^CD62L^+^, T_CM_ and CD3^+^CD8^+^CD44^+^CD62L^−^, T_EM_) in the spleen. And it was shown that the memory T cells were significantly increased in PEG‐CuP‐COF@∆St + US group with an average proportion of 64.40% T_CM_ and 18.36% T_EM_, suggesting a long‐term effect against bone metastases (Figure 8H,I; Figure S30, Supporting Information). Collectively, these results confirmed that the treatment strategy of PEG‐CuP‐COF@∆St + US could effectively facilitate pyroptosis for eliminating primary tumors while simultaneously inhibiting distant tumors and bone metastases.

## 3 Discussion

The activation of pyroptosis has emerged as a highly promising strategy for antitumor therapy due to its unique ability to trigger robust antitumor immune responses. This occurs primarily through the release of sufficient DAMPs, which enhance tumor immunogenicity and promote immune cell recruitment.^[^ ^1^, ^2^ ^]^ However, the challenge lies in effectively inducing targeted pyroptosis in tumor cells while minimizing off‐target effects, which has driven ongoing research to explore the convergence of pyroptosis, nanotechnology, chemotherapy, and other therapeutic modalities.^[^ ^43^ ^]^ Although certain chemotherapeutic agents have been shown to induce pyroptosis, their efficacy is often compromised by issues such as drug resistance and insufficient induction of pyroptotic pathways.^[^ ^44^, ^45^ ^]^ To overcome these limitations, various nanoparticles have been engineered to promote pyroptosis. Despite these advancements, many of the previously developed nanoparticles have struggled with the issue of non‐specificity.^[^ ^13^, ^26^, ^46^ ^]^ Their inability to selectively target tumor tissues has led to unintended pyroptosis in healthy cells, reducing the overall therapeutic efficacy and increasing potential side effects. Our study addresses these challenges by developing a novel PEG‐CuP‐COF@ΔSt nanosystem, which combines the strengths of pyroptosis‐inducing nanozymes (PEG‐CuP‐COF) with tumor‐targeting bacteria (ΔSt). This system not only enhances tumor specificity but also provides a unique platform for combining pyroptosis induction with sono‐catalytic therapy and immunotherapy.

Compared to other nanotechnology‐based approaches for inducing pyroptosis, our PEG‐CuP‐COF@ΔSt nanosystem offers several key advantages.^[^ ^13^, ^26^ ^]^ First, its tumor‐targeting ability significantly reduces the risk of off‐target effects, which is a major limitation of many previously reported nanoparticle systems. Second, the integration of sono‐catalytic therapy with pyroptosis activation creates a synergistic effect that enhances the overall therapeutic outcome, which many existing therapies do not achieve. Moreover, the involvement of immunotherapy further amplifies the immune response against tumors, providing a multifaceted approach to cancer treatment. However, despite the promising results, it is important to recognize the potential limitations of this approach. One significant challenge is the immunosuppressive nature of the tumor microenvironment, which may hinder the full efficacy of the combinatorial sono‐catalytic immunotherapy.^[^ ^47^, ^48^ ^]^ Further research should focus on strategies to modulate the tumor immune microenvironment, potentially through the use of immune checkpoint inhibitors or other immunomodulatory agents, to enhance the therapeutic potential of this nanosystem.

In summary, the PEG‐CuP‐COF@ΔSt nanosystem represents a novel and effective strategy for the treatment of prostate cancer by leveraging the combined effects of nanotechnology, pyroptosis induction, sono‐catalytic therapy, and immunotherapy. This innovative approach not only overcomes some of the key limitations of previous methods but also opens up new possibilities for treating a wide range of cancers. Future studies should aim to further optimize this system and explore its application in other tumor types, while addressing the challenges posed by the tumor immune microenvironment to maximize its therapeutic benefits.

## 4 Experimental Section

### Ethical Issues

All animal experiments comply with the guidelines of the Regional Ethics Committee for Animal Experiments and were approved by the Laboratory Animal Center of Shanghai Tenth People's Hospital (Approval Number: SHDSYY‐2024‐Y3778).

### Materials and Reagents

Meso‐tetrakis (4‐formylphenyl) porphyrin (P), terephthalic dihydrazide and 5,5′‐dithiobis (2‐nitrobenzoic acid) (DTNB) were purchased from Aladdin (Shanghai, China). [Amino (polyethylene glycol) 2000] (PEG‐NH_2_) was obtained from Xi'an Ruixi Biological Technology Co. (Xian, China). Hydrogen peroxide (H_2_O_2_) was purchased from Sinopharm Chemical Reagent Co., Ltd. (Shanghai, China). 1,3‐diphenylisobenzofuran (DPBF) and singlet oxygen sensor green (SOSG) was purchased from Sigma‐Aldrich (St. Louis, MO, USA). 5,5‐dimethyl‐1‐pyrroline‐N‐oxide (DMPO), dimethyl formamide (DMF), n‐butanol, o‐dichlorobenzene, acetic acid, ethanol and 4,4′‐Diamino‐3,3′,5,5′‐tetramethylbiphenyl (TMB) were obtained from Adamas‐beta Inc. (Shanghai, China). Live/Dead Cell Double Staining Kit, 2,7‐dichlorodihydrofluorescein diacetate (DCFH‐DA), cell counting kit‐8 (CCK‐8), 2‐(4‐Amidinophenyl)‐6‐indolecarbamidine dihydrochloride (DAPI), SOD assay kit with WST‐8, the LDH Cytotoxicity Assay Kit, radio‐immunoprecipitation assay (RIPA) buffer, the BCA Protein Assay Reagent Kit, SDS‐PAGE Gel Rapid Preparation Kit, QuickBlock blocking agent and transferred to polyvinylidene difluoride (PVDF) membrane were purchased from Beyotime Co. Ltd., China. ThiolTracker Violet dye was purchased from Invirogen (California, USA). Phosphate‐buffered saline (PBS), RPMI‐1640 medium, fetal bovine serum (FBS) and penicillin/streptomycin were purchased from Gibco Life Technolgies Co., Ltd (New York, US).

### Characterization

Transmission electron microscopy (TEM) and corresponding element mapping images were acquired using a field‐emission transmission electron microscope (FEI Tecnai G2 F20) operated at an accelerating voltage of 200 kV (JEOL Company Ltd., Japan). Particle size and zeta potential were detected by Zetasizer Nanoseries (ZS90, Malvern Instrument Ltd. UK). UV‐vis absorption spectra were recorded by an ultraviolet‐visible‐nearinfrared (UV‐vis‐NIR) scanning spectrophotometer (2401 PC, Varian). The Bruker Tensor II (Bruker Optics, Germany) was used to collect Fourier transform infrared (FT‐IR) spectra by a KBr method. Powder X‐ray diffraction (PXRD) characterization was performed by using a Rigaku D/max‐B II XRD instrument (Rigaku Co. Ltd., Tokyo, Japan) with 2*θ* ranging from 10 to 90° Cu Kα1. The electron spin resonance (ESR) spectrum demonstrating the quantity of **·**OH was measured by an ESR spectrometer (A200S‐95/12, Bruker, Germany) using 5,5‐dimethyl‐1‐pyrroline‐N‐oxide (DMPO) as spin‐trap agent. The fluorescent images were captured by Nikon bio‐microscope (Nikon E100, Japan) or confocal laser scanning microscopy (CLSM, CarlZeiss LSM710, ZEISS, Germany). DJO‐2776 sonicator was choosed for ultrasound irradiation in SDT.

### Preparation of COF Nanosystem

- **Synthesis of P‐COF**. Meso‐tetrakis (4‐formylphenyl) porphyrin (15 mg, 0.02 × 10^−3^ m), terephthalic dihydrazide (8 mg, 0.04 × 10^−3^ m), n‐butanol (1 mL), and o‐dichlorobenzene (1 mL) were completely dissolved in a Pyrex tube. After thorough mixing, 100 µL (6 m) of glacial acetic acid was added and the mixture was sonicated for 15 min to obtain a homogeneous solution. Then the solution was quickly frozen at 77 K (liquid N_2_ bath) and evacuated to an internal pressure of 30 mTorr. After natural warm up to room temperature, the tube was heated at 120 °C for 3 days. The product (P‐COF) was isolated by centrifuge (8000 rpm), washed with tetrahydrofuran until the supernatant was colorless.
- **Synthesis of CuP‐COF**. Hundred milligrams P‐COF, 15 mL DMF, and 75 mg CuCl_2_ • 2H_2_O were reacted in a flask at 120 °C for 1 h. Then 0.5 g NaCl was added and refluxed at 120 °C for 12 h. The product (CuP‐COF) was collected by centrifuge (8000 rpm), washed with DMF (3 times) and deionized water (3 times).
- **Synthesis of PEG‐CuP‐COF**. Fifty milligrams CuP‐COF, 100 mg PEG‐NH_2_ (MW = 2000), and 100 mL PBS were stirred for 24 h. The resulting mixture was centrifuged at 1500 rpm for 30 min to discard un‐isolated nanoparticles, and then centrifuged at 10 000 rpm for 20 min to obtain the isolated PEG‐CuP‐COF samples.
- **Synthesis of PEG‐CuP‐COF@ΔSt**. PEG‐CuP‐COF nanoparticles were stirred with Attenuated Salmonella Typhimurium strain VNP20009 (ΔSt) (1.0 mg PEG‐CuP‐COF/10^6^ CFU ΔSt) in 1 mL PBS (added with 1.15 mg EDC and 1.3 mg NHS) for 0.5 h. Then PEG‐CuP‐COF@ΔSt were obtained by centrifuge (3000 rpm, 5 min) and washed with PBS three times.

### Reactive Oxygen Species (ROS) Production Ability of PEG‐CuP‐COF In Vitro

  - DPBF (20 µg mL^−1^), a ^1^O_2_ indicator, was mixed with various solution samples including P (50 µg mL^−1^), P+US, PEG‐P‐COF (50 µg mL^−1^), PEG‐P‐COF+US, PEG‐CuP‐COF (50 µg mL^−1^), PEG‐CuP‐COF+US, PEG‐CuP‐COF@ΔSt+US. Then US irradiation (1.0 MHz, 1.0 W cm^−2^, 50% duty cycle) was performed on solution samples containing US. The UV–vis absorption spectra of DPBF at the wavelength of 425 nm every 1 min (0, 1, 2, 3, 4, 5 min) in various solution samples was recorded.
  - SOSG (5 × 10^−6^ m), also a ^1^O_2_ indicator, was mixed with PEG‐CuP‐COF (50 µg mL^−1^). Then the mixed solution was exposed to US irradiation (1.0 MHz, 1.0 W cm^−2^, 50% duty cycle, 5 min). The fluorescence spectral change of SOSG was recorded by fluorescence spectrophotometer (λ_ex_ = 488 nm).
  - DCFH (50 × 10^−9^ m) and P, PEG‐P‐COF, PEG‐CuP‐COF (50 µg mL^−1^) were placed together, respectively, and then treated with US irradiation (1.0 MHz, 1.0 W cm^−2^, 50% duty cycle) for various durations (0, 1, 2, 3, 4, 5 min). Fluorescence spectrum of ROS generation was measured every 1 min (0, 1, 2, 3, 4, 5 min) by a multifunctional microplate reader (λ_ex_ = 488 nm).

### Multienzyme‐Mimicking Activities

- **Superoxide dismutase (SOD)‐mimicking activity**: The ability of PEG‐CuP‐COF scavenging superoxide anion radical (O_2_^−•^) was evaluated by measuring the inhibition of water‐soluble formazan formation through WST‐8 using a SOD assay kit according to the supplier's protocols. Briefly, O_2_^−•^ can react with WST‐8 catalyzed by Xanthine Oxidase (XO) to produce formazan. Meanwhile, SOD can catalyze the dismutation of O_2_^−•^. So, the SOD activity was negatively correlated with the amount of formazan and can be measured by colorimetric analysis of formazan. Therefore, to assess the SOD‐mimicking activity, PEG‐P‐COF@ΔSt (50, 75, 100 µg mL^−1^), PEG‐P‐COF@ΔSt + US (1.0 MHz, 1.0 W cm^−2^, 50% duty cycle, 5 min), PEG‐CuP‐COF@ΔSt (50, 75, 100 µg mL^−1^), and PEG‐CuP‐COF@ΔSt + US was separately added to the solution containing WST‐8, XO and xanthine, then continuing reacting at 37 ^°^C for 30 min. Finally, the absorption changes of water‐soluble formazan in the acquired supernatant were spectrophotometrically monitored at 450 nm using UV–vis spectroscopy.
- **Peroxidase (POD)‐mimicking activity**: The POD‐mimicking activity of PEG‐CuP‐COF was evaluated by the absorption peak of TMB at 654 nm according to the oxidation color development. In brief, H_2_O_2_ (0.1 mm), PEG‐P‐COF (100 µg mL^−1^), PEG‐CuP‐COF (100 µg mL^−1^), PEG‐CuP‐COF+H_2_O_2_, PEG‐CuP‐COF+H_2_O_2_+US (1.0 MHz, 1.0 W cm^−2^, 50% duty cycle, 5 min) and PEG‐CuP‐COF@ΔSt+H_2_O_2_+US was separately mixed with TMB (0.2 µm), and then the optical absorbance of the acquired liquid was detected at 654 nm using a microplate reader every 5 min. The UV–vis absorption spectra in different reaction systems were also recorded.
- **Glutathione peroxidase (GPx)‐mimicking activity**: To detect the GPx‐mimicking activity, H_2_O_2_ (0.1 mm), PEG‐P‐COF (100 µg mL^−1^), PEG‐CuP‐COF (100 µg mL^−1^), PEG‐CuP‐COF+H_2_O_2_, PEG‐CuP‐COF+H_2_O_2_+US (1.0 MHz, 1.0 W cm^−2^, 50% duty cycle, 5 min) and PEG‐CuP‐COF@ΔSt+H_2_O_2_+US was separately reacted with GSH (1 mm) at 37 °C for 3 h. Then 20 µL DTNB (3 mg mL^−1^) and 100 µL supernatant which removing the nanoparticles by centrifuge was added to a 96‐well plate, and further incubated at 37 °C for 30 min. Finally, the optical absorbance of the acquired supernatant was measured at 412 nm using a microplate reader. And UV–vis absorption spectra in different reaction systems were also recorded.

### Detection of •OH Generation by ESR

ESR spectroscopy was used to detect the •OH generation of PEG‐CuP‐COF using DMPO as trapping agent. PEG‐CuP‐COF (100 µg mL^−1^) was dispersed into PBS buffer (pH4.5) with or without H_2_O_2_ (10 mm)/US irradiation (1.0 MHz, 1.0 W cm^−2^, 50% duty cycle, 5 min). The signals of •OH were detected with the addition of DMPO (25 mm).

### Bacteria Culture

Attenuated Salmonella Typhimurium strain VNP20009 (ΔSt) was cultured in culture tubes containing Luria‐Bertani (LB) broth under anaerobic condition in an oscillation incubator at 37 °C. ΔSt was collected by centrifuge (4 °C, 2500 *g*, 5 min) and then suspended to 1 × 10^6^ CFU mL^−1^.

### Bacteria Viability

The bacteria (1 × 10^6^ CFU mL^−1^) were stirred with various concentrations (0, 0.5, 1.0, 1.5, and 2.0 mg mL^−1^) of PEG‐CuP‐COF for 12 h. After that the old culture medium was replaced with PBS containing 5% CCK‐8, and the bacterial viability was assessed using a microplate reader (λ = 450 nm, Molecular Devices, CA, USA). Meanwhile, the bacteria samples stirred with various concentrations of PEG‐CuP‐COF at three time points (0, 6, and 12 h) were plated on LB plates and the bacterial titer (CFU mL^−1^) was presented by the ratio of colony counts and dilution multiple.

### Detection of pH Value

The pH values of LB medium and LB medium containing PEG‐CuP‐COF@ΔSt were measured at different time points (0, 0.5, 1, 2, 4, 8, and 12 h) by a pH meter (INESA Scientific Instrument Co. PHSJ‐6L).

### Cell Culture

Three kinds of cells were used in vitro. The Alpha mouse liver 12 (AML‐12) cells, normal mouse prostate epithelial cells (PrECs), and prostate cancer cells (RM‐1) were obtained from the Cell Bank of Shanghai Institutes for Biological Sciences, Chinese Academy of Sciences (Shanghai, China). All cells were cultured in RPMI‐1640 (or DMEM) medium with 10% fetal bovine serum (FBS) and 1% penicillin/streptomycin at 37 °C in an incubator with 5% CO_2_.

### Cellular Uptake Detection

RM‐1 cells were seeded into CLSM‐specific culture dishes (1 × 10^5^ per well) and cultured overnight. The old medium was replaced by Cy5.5‐labeled PEG‐CuP‐COF (30 µg mL^−1^). After co‐incubation for 0, 0.5, 1, 2, 4, and 6 h, the cells were washed with PBS for three times and stained with DAPI for 20 min. Then the cellular uptake of PEG‐CuP‐COF nanoparticles was visualized by CLSM.

### Cell Viability and SDT‐Mediated Cytotoxicity of PEG‐CuP‐COF

To detect the cytotoxicity of PEG‐CuP‐COF, RM‐1, PrECs, and AML‐12 cells were respectively seeded in 96‐well plates (1 × 10^4^ per well) and cultured overnight for attachment. Then the cells were co‐incubated with various concentrations (0, 10, 20, 30, 40, and 50 µg mL^−1^) of PEG‐CuP‐COF for 8 h. Additionally, to detect the SDT effect, US irradiation (1.0 W cm^−2^, 1.0 MHz, 50% duty cycle, 5 min) was performed on RM‐1 cells. And the cell viability was measured by CCK‐8 assay using a microplate reader (λ = 450 nm, Molecular Devices, CA, USA).

### Cell Live/Death Detection

For live/dead cell staining analysis, RM‐1 cells were cultured in 6‐well plates (1 × 10^5^ per well) overnight for attachment and then given different treatments including control (without any treatment), H_2_O_2_ (100 × 10^−6^ m), US only (1.0 MHz, 1.0 W cm^−2^, 50% duty cycle, 5 min), PEG‐CuP‐COF (30 µg mL^−1^), PEG‐CuP‐COF+H_2_O_2_, PEG‐CuP‐COF+US, and PEG‐CuP‐COF+H_2_O_2_+US. Then cells were washed with PBS and stained with Calcein‐AM/PI for 15 min. Finally, the live and dead conditions of RM‐1 cells were observed by fluorescence microscope, respectively.

### Cell Apoptosis Analysis

For cell apoptosis analysis using FCM, RM‐1 cells were seeded in a 6‐well plate (1 × 10^5^ per well) and treated the same way as described above. Following trypsin‐mediated dissociation, the cells were gently collected and stained by an Annexin V‐FITC/PI Kit according to standard processes. The apoptosis analysis was then conducted by FACS Canto II.

### Intracellular ROS Detection

RM‐1 cells were seeded in CLSM‐specific culture dishes at a density of 1 × 10^5^ and incubated at 37 °C overnight, then the culture dishes were randomly divided into seven groups: (G1) Control, (G2) H_2_O_2_ (100 × 10^−6^ m), (G3) US only, (G4) PEG‐CuP‐COF (30 µg mL^−1^), (G5) PEG‐CuP‐COF+H_2_O_2_, (G6) PEG‐CuP‐COF+US, and (G7) PEG‐CuP‐COF+H_2_O_2_+US. After continuing incubation with various samples for 8 h, the cells were washed with PBS and incubated with DCFH‐DA probe at 37 °C for 30 min. Then US irradiation (1.0 MHz, 1.0 W cm^−2^, 50% duty cycle, 5 min) was performed on designated groups. Subsequently, the intracellular ROS generation in different groups was observed by CLSM with Ex/Em of 488/525 nm.

### Intracellular GSH Consumption

The cells were treated the same way as mentioned above and then incubated with 20 µm ThiolTracker Violet dye at 37 °C for 30 min. The GSH in different groups was observed by CLSM with Ex/Em of 404/526 nm.

### Lactate Dehydrogenase (LDH) and Interleukin‐1β (IL‐1β) Release Detection

For LDH release detection, RM‐1 cells were seeded into 96‐well plates (1 × 10^4^ per well) and incubated overnight at 37 °C for attachment. Then the cells were randomly treated with the following conditions: (G1) Control, (G2) H_2_O_2_ (100 × 10^−6^ m), (G3) US only, (G4) PEG‐CuP‐COF (30 µg mL^−1^), (G5) PEG‐CuP‐COF+H_2_O_2_, (G6) PEG‐CuP‐COF+US, and (G7) PEG‐CuP‐COF+H_2_O_2_+US. After continuing incubation for 8 h, US irradiation (1.0 MHz, 1.0 W cm^−2^, 50% duty cycle, 5 min) was performed on the designated groups. Then the cells were incubated at 37 °C for another 6 h. After removing the nanoparticles by centrifuge, 100 µL supernatant from each well was taken and added to a new 96‐well plate. Then 50 µL LDH detection working fluid was added to co‐incubate for 30 min. Finally, the optical absorbance of the acquired samples at 490 nm was detected by a microplate reader. For IL‐1β release detection, it was performed by the IL‐1β ELISA kit according to the manufacturer's instructions.

### Western Blot

RM‐1 cells were seeded on 6‐well plates and incubated overnight. Then the cells were randomly divided into seven groups mentioned above. After different treatments, the cells were collected and lysed in lysis buffer on ice for 30 min, and then centrifuged with 12 000 rpm for 10 min. The protein concentration of the supernatant was analyzed by a BCA Protein Assay Kit. After boiling with loading buffer for 10 min, equal amounts of proteins (50 µg) were fractionated by 10% SDS‐PAGE and transferred to PVDF membranes. The membranes were then blocked with 5% milk for 1.5 h at room temperature following by washing thrice with TBST for 10 min, and incubated with primary antibodies at 4 °C overnight. The primary antibodies included: β‐actin Anti‐Rabbit Antibody (Abcam, Cat# ab8227), CRT (Affinity Biosciences, Cat# DF10202), HMGB1 (Affinity Biosciences, Cat# AF7020), Caspase‐3 (Abcam, Cat# ab184787), GSDMD (Abcam, Cat# ab209845), and GSDME (Abcam, Cat# ab215191). After incubation with the secondary antibody for 1.5 h at room temperature, the membrane was washed with TBST for three times again and visualized by chemiluminescence HRP substrate using a chemiluminescent (ECL) detection apparatus. Their expressions were also analyzed quantitatively by Image J.

### In Vivo Safety Evaluation

First, a bio‐safety evaluation of ΔSt was performed on three groups (per group, *n *= 3) by injecting different amounts of ΔSt (200 µL per mouse, 1 × 10^5^, 1 × 10^6^, and 1 × 10^7^ CFU mL^−1^) into healthy C57BL/6 mice (male, four‐ to five‐week‐old) for optimizing the dose of bacteria for subsequent application in vivo. Body weight of mice was recorded every day during day 0–30.

Second, to monitor the toxicity at different stages, the healthy C57BL/6 mice were randomly divided into five groups (per group, *n *= 3): Control, Day 1, Day 3, Day 7, and Day 30. Blood was collected on control (without any treatment) and 1, 3, 7, and 30 days after tail vein injection of PEG‐CuP‐COF@ΔSt (200 µL per mouse, 1 mL PEG‐CuP‐COF@ΔSt contained 1 × 10^6^ CFU ΔSt and 1.0 mg PEG‐CuP‐COF) for biochemical parameters and liver and kidney function parameters. The major organs (heart, liver, spleen, lung, kidney) were also harvested for H&E staining and LB agar plate count.

### In Vivo Fluorescence Bio‐Distribution Study

RM‐1 cells (1 × 10^6^) were subcutaneously implanted into the right flanks of C57BL/6 mice (male, four‐ to five‐week‐old). When the tumor volume reached ≈200 mm^3^, Cy5.5‐labeled PEG‐CuP‐COF (200 µL per mouse, 1.0 mg mL^−1^ PEG‐CuP‐COF), Cy5.5‐labeled ΔSt (200 µL per mouse, 1 × 10^6^ CFU mL^−1^ ΔSt) and Cy5.5‐labeled PEG‐CuP‐COF@ΔSt (200 µL per mouse, 1 mL PEG‐CuP‐COF@ΔSt contained 1 × 10^6^ CFU ΔSt and 1.0 mg PEG‐CuP‐COF) were intravenously injected into three groups, respectively. Then mice were visualized at continuous time points (0, 2, 4, 8, 12, 24, 48, and 72 h) using imaging system (BLT AniView100, Guangzhou Biolight Biotechnology Co., Ltd.). The major organs (heart, liver, spleen, lung, and kidney) and tumors were harvested for quantitative bio‐distribution analysis and LB agar plate counts were also performed.

### In Vivo Antitumor Efficacy

RM‐1 cells (1 × 10^6^) were subcutaneously implanted into the right flanks of C57BL/6 mice (male, four‐ to five‐week‐old) to establish tumor models. When the average tumor volume reached 75–100 mm^3^ after growth for 7 days, the mice were randomly divided into six groups (per group, *n *= 15): (G1) Control, (G2) US only, (G3) PEG‐CuP‐COF (200 µL per mouse, 1.0 mg mL^−1^ PEG‐CuP‐COF), (G4) PEG‐CuP‐COF@ΔSt (200 µL per mouse, 1 mL PEG‐CuP‐COF@ΔSt contained 1 × 10^6^ CFU ΔSt and 1.0 mg PEG‐CuP‐COF), (G5) PEG‐CuP‐COF + US, and (G6) PEG‐CuP‐COF@ΔSt + US. The PEG‐CuP‐COF and PEG‐CuP‐COF@ΔSt were intravenously injected on days 0, 2, and 4, respectively, and US irradiation (1.0 MHz, 1.0 W cm^−2^, 50% duty cycle, 5 min) was performed on the designated groups on days 1, 3, and 5, respectively. Tumor volume and body weight of mice were recorded every two days during day 0–14. The tumor volume was calculated by the following formula: volume (V, mm^3^)  =  (length × width^2^)/2. On day 6, mice (per group, *n* = 3) were sacrificed to collect tumors for terminal deoxynucleotidyl transferase‐mediated dUTP nick‐end labeling (TUNEL), calreticulin (CRT), high mobility group box 1 (HMGB1), Granzyme B (GzmB), Ki‐67, c‐Caspase‐3, CD3^+^CD4^+^, CD3^+^CD8^+^, and H&E staining, and their expressions were analyzed quantitatively by Image J. On day 14, the primary tumors (per group, *n *= 5) were harvested and photographed. And the survival rate was also evaluated from day 0 to day 30.

### In Vivo Antitumor Immunity

In order to explore the anticancer immune effect, tumor tissues, draining lymph nodes and spleens were isolated after the initial various treatments and homogenized into single‐cell suspensions. Then flow cytometry was used to analyze the proportion of immune cells which including CD3^+^CD4^+^ T cells, CD3^+^CD8^+^ T cells, CD3^+^CD4^+^FOXP3^+^ T cells, CD11c^+^CD86^+^CD80^+^ DCs. Additionally, ELISA kits were used to examine the expression levels of various cytokines including IL‐10, IL‐6, IFN‐γ, and TNF‐α in RM‐1 tumor tissues.

### RNA Sequencing

RM‐1 tumor‐bearing mice (tumor volumes ranging from 75 to 100 mm^3^) were randomly divided into two groups (per group, *n *= 3): control group (without any treatment), treated group (PEG‐CuP‐COF@ΔSt + US). Then the treated group was given three times of intravenously injection followed by US irradiation as mentioned above. To prevent RNA degradation, tumor tissues in two groups were collected for RNA‐sequencing at 4 h after treatment. The tissues were subsequently sequenced and analyzed by Shanghai OE Biotech Co. (Shanghai, China).

### In Vivo Distant Tumor Inhibition

For the primary tumor development, RM‐1 cells (1 × 10^6^) were subcutaneously injected into the right flank of each mouse. When the primary tumor volume reached 75–100 mm^3^, the distant tumor models were established by injecting RM‐1 cells (1 × 10^6^) into the left flanks. Then the mice were randomly divided into six groups (per group, *n *= 10) including: (G1) Control, (G2) US only, (G3) PEG‐CuP‐COF, (G4) PEG‐CuP‐COF@ΔSt, (G5) PEG‐CuP‐COF+US, and (G6) PEG‐CuP‐COF@ΔSt+US. Subsequently, the primary tumors were subjected to the above treatments, whereas no treatment was performed to the distant tumors. The bilateral tumor volumes and body weight of mice were recorded every two days during days 0–14. Additionally, the mice (per group, *n *= 3) were also euthanized for immunofluorescence staining of distant tumor tissues to explore T cells with CD3^+^CD4^+^/CD3^+^CD8^+^ markers. On day 14, the bilateral tumors (per group, *n *= 5) were harvested and photographed.

### In Vivo Anti‐Bone Metastasis Efficacy

RM‐1 tumor‐bearing mice with tumor volume 75–100 mm^3^ were randomly divided into six groups (per group, *n *= 10): (G1) Control, (G2) US only, (G3) PEG‐CuP‐COF, (G4) PEG‐CuP‐COF@ΔSt, (G5) PEG‐CuP‐COF+US, and (G6) PEG‐CuP‐COF@ΔSt+US. Subsequently, the tumors were administrated to the mentioned treatments above. Then the tumors were surgically removed after 14 days. On day 28, the bone metastasis tumor models were established as following. The femur and tibia of the right hind limb were bent at an angle of 90°, and 5 × 10^4^ RM‐1 cells labeled with luciferase were injected by puncturing into the bone marrow cavity along the long axis of the tibia for each mouse. Tumors formed within one week. The percent of memory T cells in spleens of each group (per group, *n *= 5) was analyzed by flow cytometry and stained with anti‐CD3‐FITC, anti‐CD8‐APC, anti‐CD4‐PE, anti‐CD44‐PE, and anti‐CD62L‐PE‐Cy5 antibodies. The immune T cells included central memory T cells (CD3^+^CD4^+^CD44^+^CD62L^+^, T_CM_) and effector memory T cells (CD3^+^CD8^+^CD44^+^CD62L^−^, T_EM_). Finally, right hind limb tibia of mice in various groups were harvested for H&E staining.

### Statistical Analysis

All statistical analyses were conducted using Graphpad Prism (version 9.0.0, GraphPad Software, San Diego, California, USA). Ordinary one‐way ANOVA was performed for comparison between multiple groups, and two‐tailed student's t‐test was used between two groups. Animal survival was calculated by Kaplan‐Meier method, and *P* value was obtained by the log‐rank test. Data were presented as Mean ± SD. ^****^ *P* < 0.0001, ^***^ *P* < 0.001, ^**^ *P* < 0.01, ^*^ *P* < 0.05, ns: no significance.

## Conflict of Interest

The authors declare no conflict of interest.

## Author Contributions

Y.Y.L., L.H.X., and Y.T.L. contributed equally to this work. Y.Y.L., L.H.X., Y.T.L., and Y.F.Z. designed the research strategy and experiments. Y.T.L. and S.Z. synthesized and characterized the nanoparticles. S.Y.L., Y.Z., H.S., H.L., D.D., B.G.Z., and B.B.Y. conducted in vitro and in vivo experiments. Y.Y.L., L.H.X., and Y.T.L. wrote the paper. H.H.Y., H.X.X., and Y.F.Z. supervised the whole process. All authors discussed the experimental procedures and results.

## Supporting information



Supporting Information

## Data Availability

The data that support the findings of this study are available from the corresponding author upon reasonable request.
