# Supplementary material for: Bacteria‐Mediated Tumor‐Targeting Delivery of Multienzyme‐Mimicking Covalent Organic Frameworks Promoting Pyroptosis for Combinatorial Sono‐Catalytic Immunotherapy
Source: Adv Sci (Weinh). 2024 Nov 4;11(47):2407133. doi: 10.1002/advs.202407133 (PMC11653599; doi:10.1002/advs.202407133)
Supplement: Supplementary file 1 — Supporting Information [file ADVS-11-2407133-s001.pdf]

ADVANCED SCIENCE
Open Access

Supporting Information

for *Adv. Sci.*, DOI 10.1002/advs.202407133

Bacteria-Mediated Tumor-Targeting Delivery of Multienzyme-Mimicking Covalent Organic Frameworks Promoting Pyroptosis for Combinatorial Sono-Catalytic Immunotherapy

Yunyun Liu, Lihua Xiang, Yitong Li, Shen Zhang, Ying Zhang, Hui Shi, Hui Liu, Dou Du, Bangguo Zhou, Beibei Ye, Shaoyue Li, Haohao Yin, Huixiong Xu* and Yifeng Zhang**

Supporting Information

Bacteria-mediated tumor-targeting delivery of multienzyme-mimicking covalent organic frameworks promoting pyroptosis for combinatorial sono-catalytic immunotherapy

Yunyun Liu [1,2,3#], Lihua Xiang [1,2,3#], Yitong Li [1,2,3#], Shen Zhang [1,2,3], Ying Zhang [1,2,3], Hui Shi [1,2,3], Hui Liu [1,2,3], Dou Du [1,2,3], Bangguo Zhou [1,2,3], Beibei Ye [1,2,3], Shaoyue Li [1,2,3], Haohao Yin [4*], Huixiong Xu [4*] & Yifeng Zhang [1,2,3*]

[1] Department of Medical Ultrasound, Center of Minimally Invasive Treatment for Tumor, Shanghai Tenth People's Hospital, School of Medicine, Tongji University, Shanghai 200072, P. R. China.

[2] Ultrasound Research and Education Institute, Clinical Research Center for Interventional Medicine, Shanghai Tenth People's Hospital, School of Medicine, Tongji University, Shanghai 200072, P. R. China.

[3] Shanghai Engineering Research Center of Ultrasound Diagnosis and Treatment, Shanghai 200072, P. R. China.

[4] Department of Ultrasound, Zhongshan Hospital, Institute of Ultrasound in Medicine and Engineering, Fudan University, Shanghai 200032, P. R. China.

[#]These authors contributed equally: Yunyun Liu, Lihua Xiang, Yitong Li.

*Corresponding author email: yin.haohao@zs-hospital.sh.cn; xu.huixiong@zs-hospital.sh.cn; zhangyifeng@tongji.edu.cn

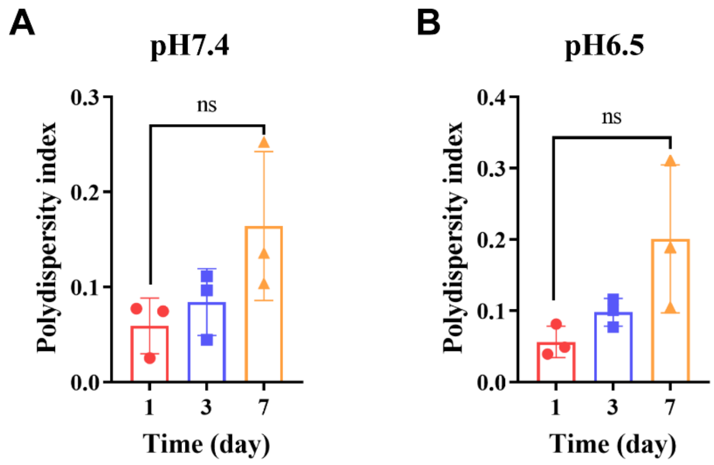

Figure S1. A) The polydispersity index (PDI) of PEG-CuP-COF in neutral condition (pH7.4). B) The polydispersity index (PDI) of PEG-CuP-COF in acidic condition (pH6.5). Data are presented as Mean ± SD. $^{****}P < 0.0001$, $^{***}P < 0.001$, $^{**}P < 0.01$, $^{*}P < 0.05$, ns: no significance.

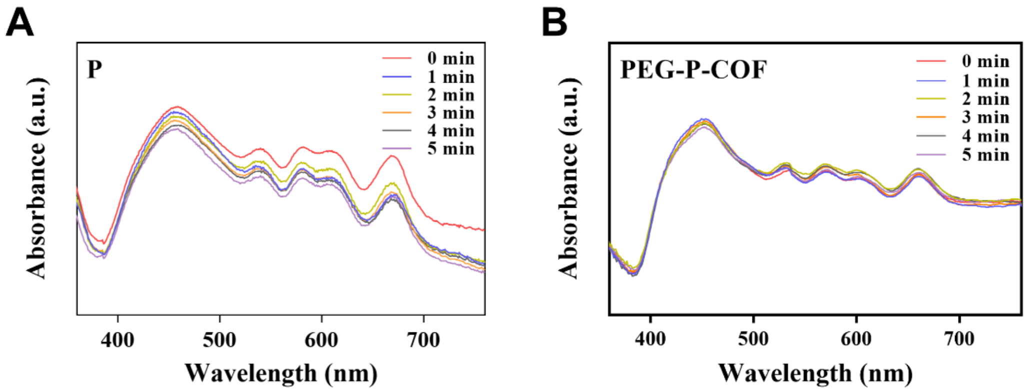

Figure S2. A) UV-vis absorption spectra of P under US irradiation (1.0 W cm^{-2}, 1.0 MHz, 50% duty cycle). B) UV-vis absorption spectra of PEG-P-COF under US irradiation (1.0 W cm^{-2}, 1.0 MHz, 50% duty cycle).

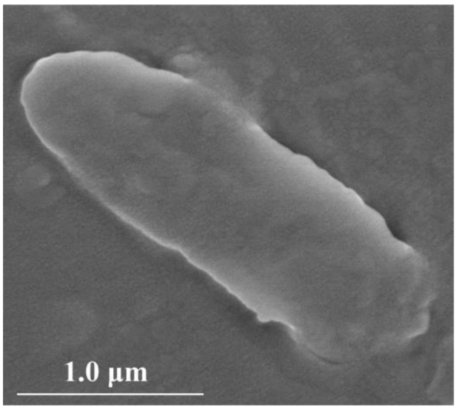

Figure S3. Representative scanning electron microscope (SEM) image of ΔSt.

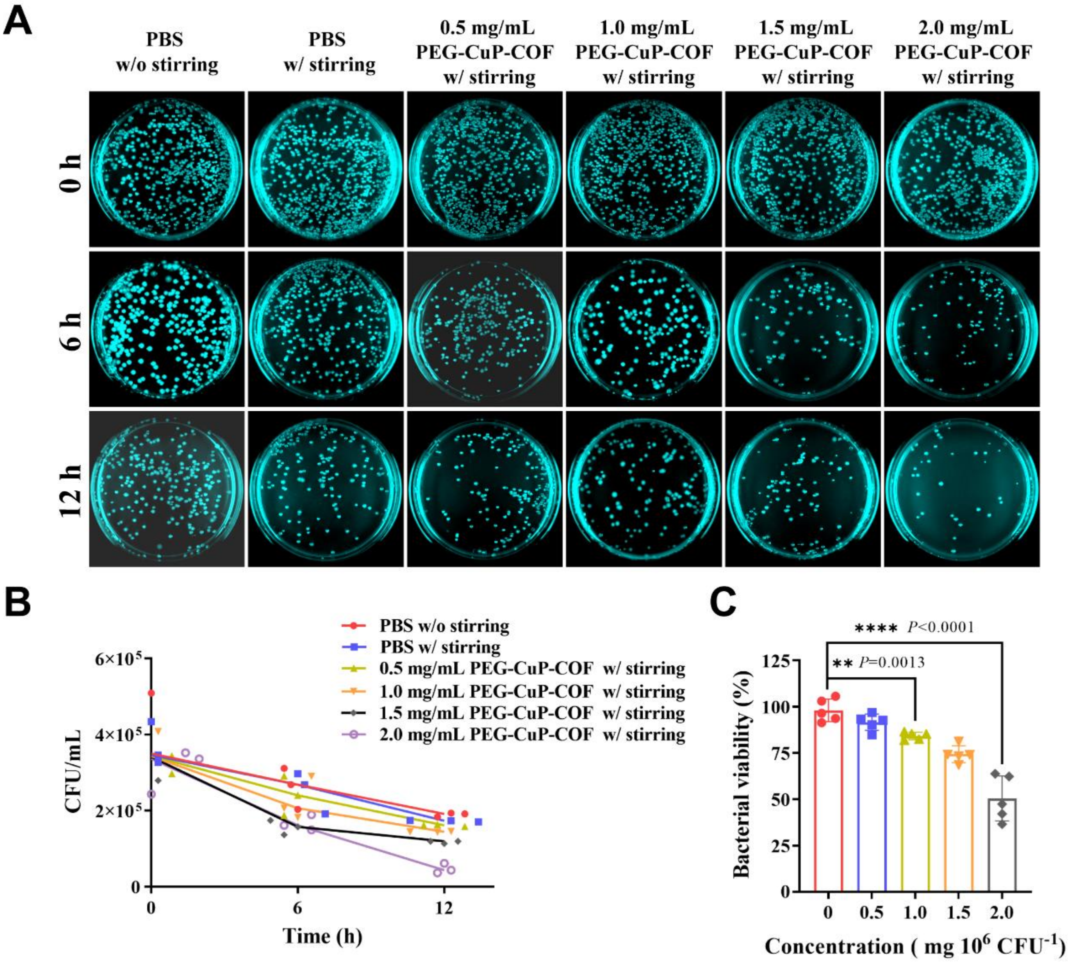

Figure S4. A) Representative photographs of LB agar plates and B) corresponding CFU quantification of bacterial activity with various concentrations of PEG-CuP-COF for different times (0, 6 and 12 h). C) Bacterial viability of ΔSt treated with different concentrations of PEG-CuP-COF for 12 h. Data are presented as Mean ± SD. $^{****}P < 0.0001$, $^{***}P < 0.001$, $^{**}P < 0.01$, $^{*}P < 0.05$, ns: no significance.

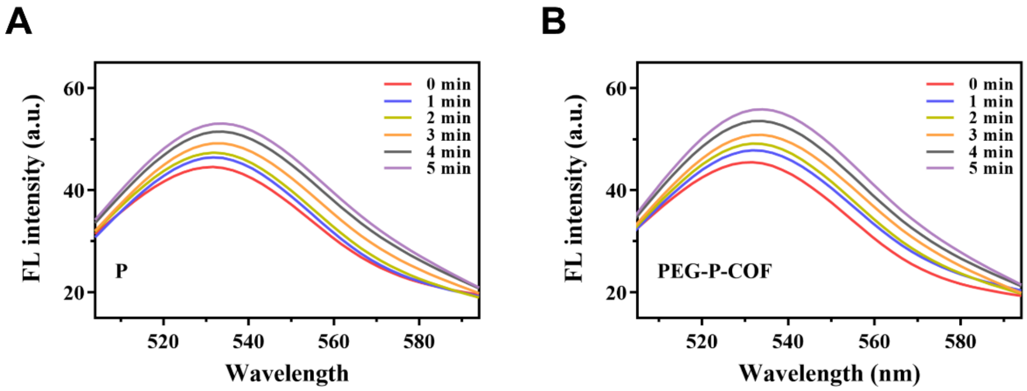

Figure S5. A) DCF fluorescence spectra of P under US irradiation (1.0 W cm^{-2}, 1.0 MHz, 50% duty cycle). B) DCF fluorescence spectra of PEG-P-COF under US irradiation (1.0 W cm^{-2}, 1.0 MHz, 50% duty cycle).

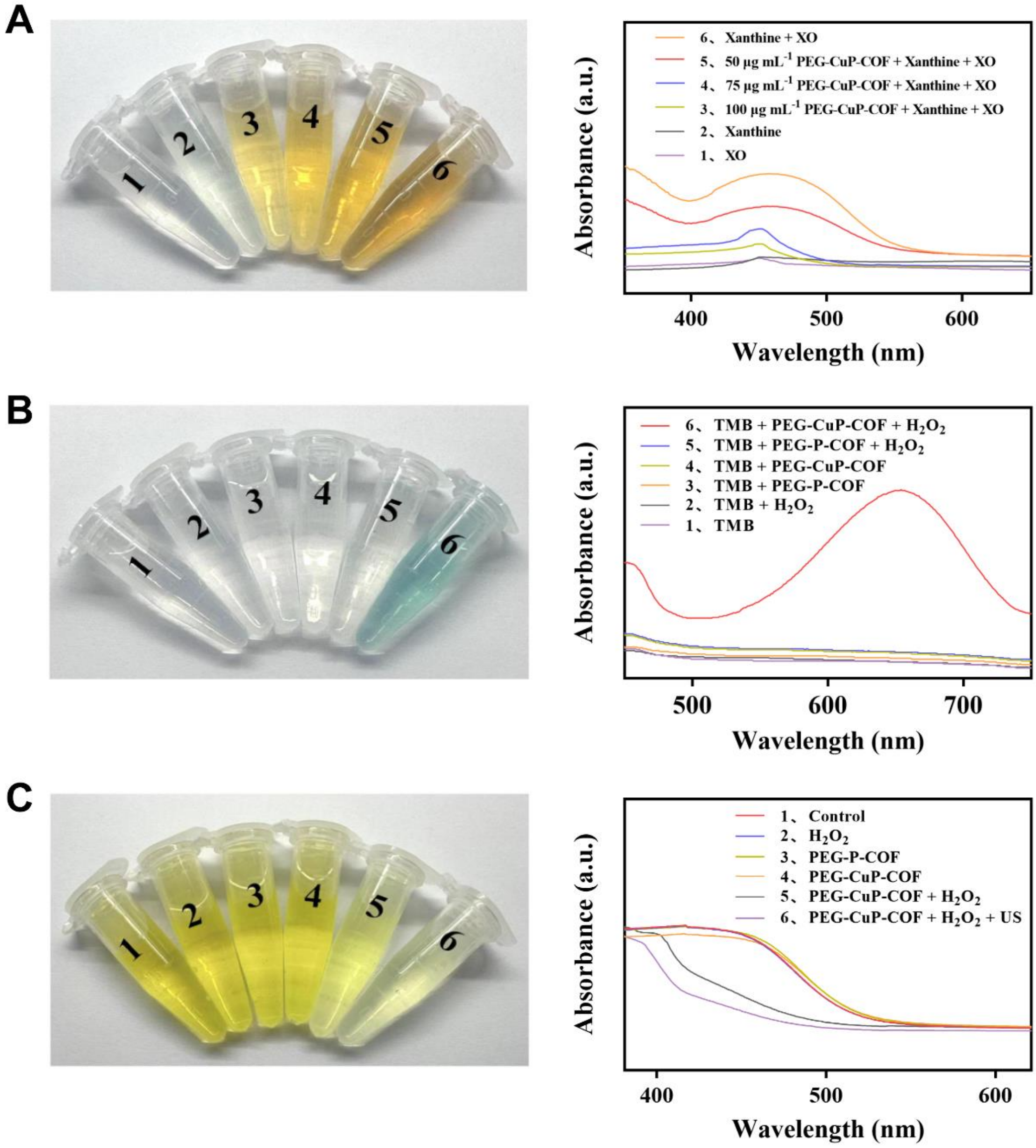

Figure S6. A) SOD-mimicking activity. Photography and UV-vis absorption spectra of the mixture of (1) Xanthine Oxidase (XO), (2) xanthine, (3) 100 μg/mL PEG-CuP-COF + xanthine + XO, (4) 75 μg/mL PEG-CuP-COF + xanthine + XO, (5) 50 μg/mL PEG-CuP-COF + xanthine + XO and (6) xanthine + XO. B) POD-mimicking activity. Photography and UV-vis absorption spectra of the mixture of (1) TMB, (2) TMB + H_2O_2, (3) TMB + PEG-P-COF, (4) TMB + PEG-CuP-COF, (5) TMB + PEG-P-COF + H_2O_2 and (6) TMB + PEG-CuP-COF + H_2O_2. C) GPx-mimicking activity. Photography and UV-vis absorption spectra of the mixture of (1) Control, (2) H_2O_2, (3) PEG-P-COF, (4) PEG-CuP-COF, (5) PEG-CuP-COF + H_2O_2 and (6) PEG-CuP-COF + H_2O_2 + US (1.0 W cm^{-2}, 1.0 MHz, 50% duty cycle, 5 min).

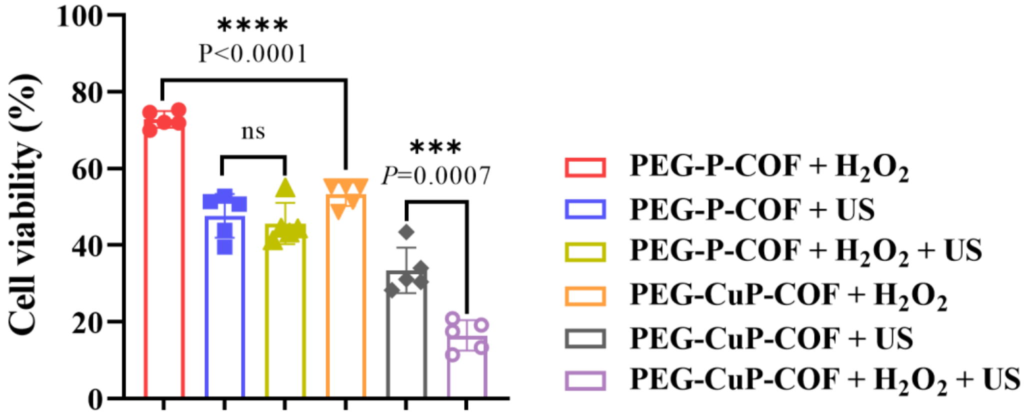

Figure S7. Viability of RM-1 cells treated under different conditions. Data are presented as Mean ± SD. $^{****}P < 0.0001$, $^{***}P < 0.001$, $^{**}P < 0.01$, $^{*}P < 0.05$, ns: no significance.

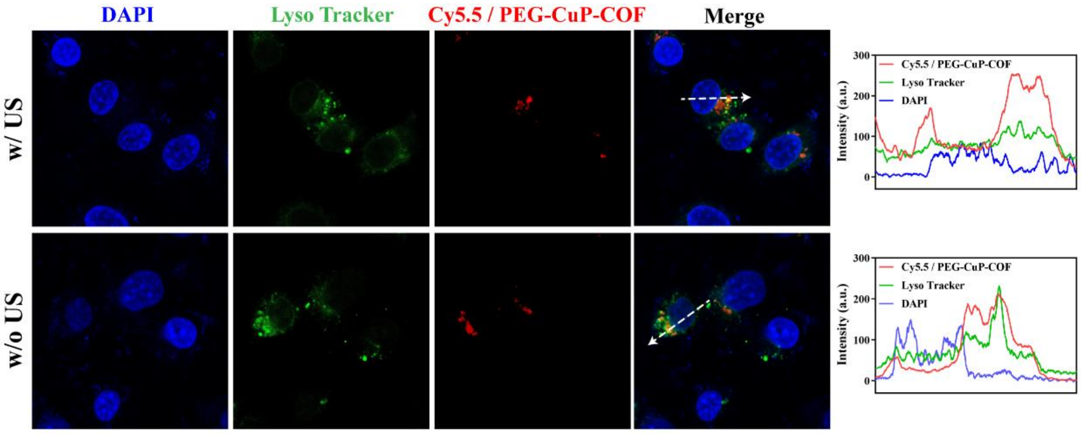

Figure S8. CLSM images and corresponding mean fluorescence intensity of RM-1 cells cultured with Cy5.5-labeled PEG-CuP-COF (with or without US irradiation).

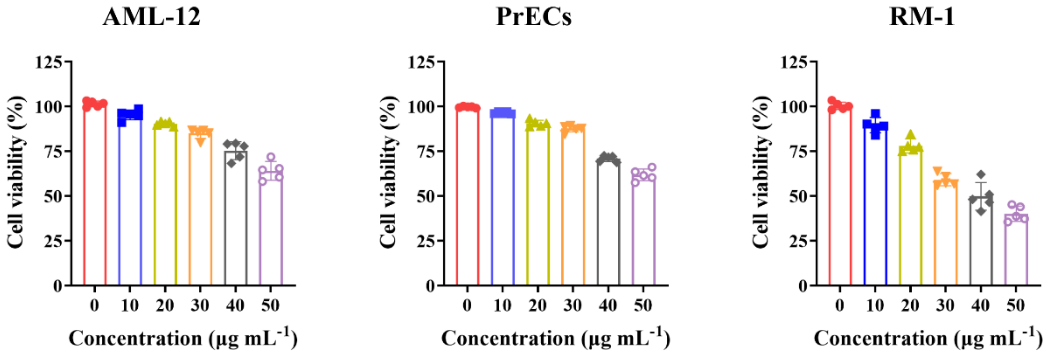

Figure S9. Viability of various cells after incubation with different concentrations of PEG-CuP-COF for 8 h. Data are presented as Mean ± SD.

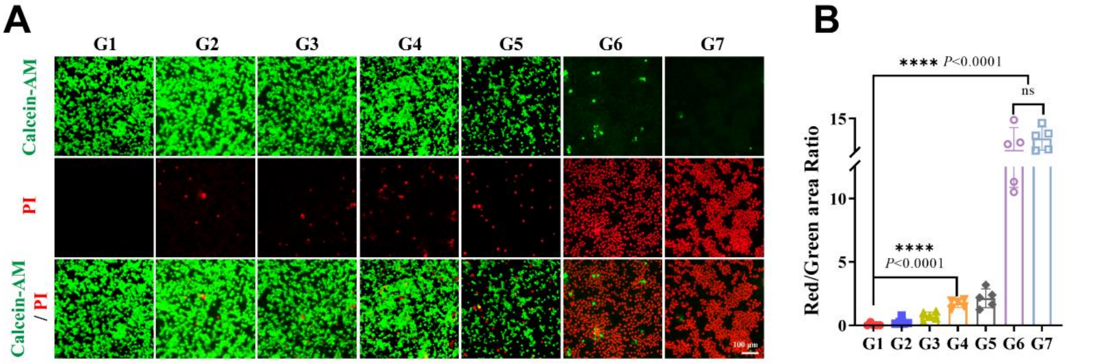

Figure S10. A) Representative fluorescence images of Calcein-AM/PI staining and B) corresponding fluorescence intensity for different groups. G1: Control, G2: H_2O_2, G3: US only, G4: PEG-CuP-COF, G5: PEG-CuP-COF + H_2O_2, G6: PEG-CuP-COF + US, G7: PEG-CuP-COF + H_2O_2 + US. Data are presented as Mean ± SD. $^{****}P < 0.0001$, $^{***}P < 0.001$, $^{**}P < 0.01$, $^{*}P < 0.05$, ns: no significance.

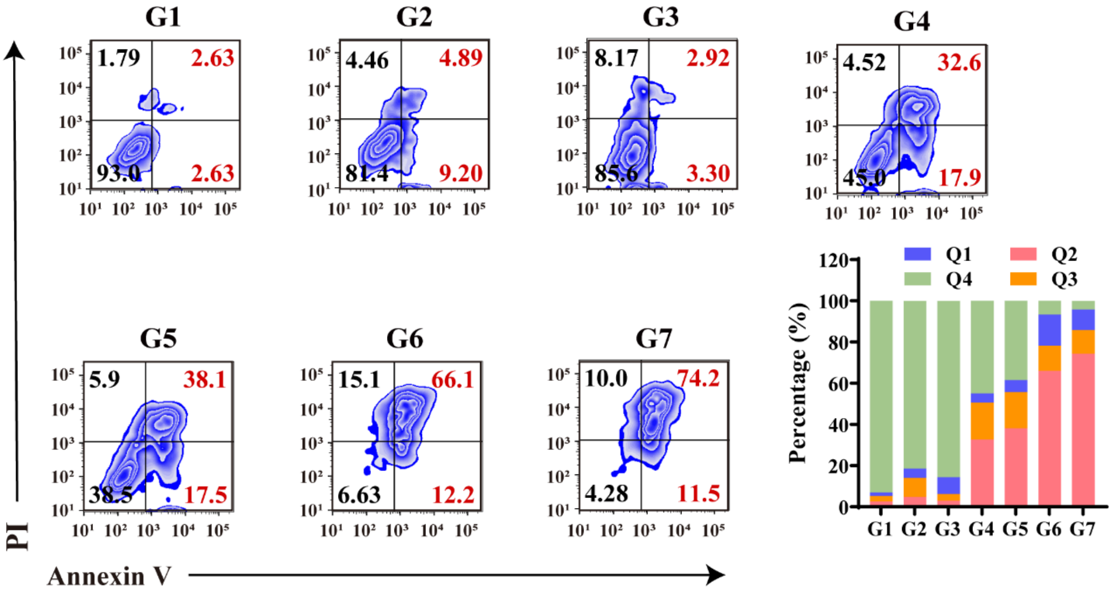

Figure S11. Annexin V-FITC and PI staining assays and the corresponding semi-quantitative analysis of RM-1 cells after different treatments. G1: Control, G2: H_2O_2, G3: US only, G4: PEG-CuP-COF, G5: PEG-CuP-COF + H_2O_2, G6: PEG-CuP-COF + US, G7: PEG-CuP-COF + H_2O_2 + US.

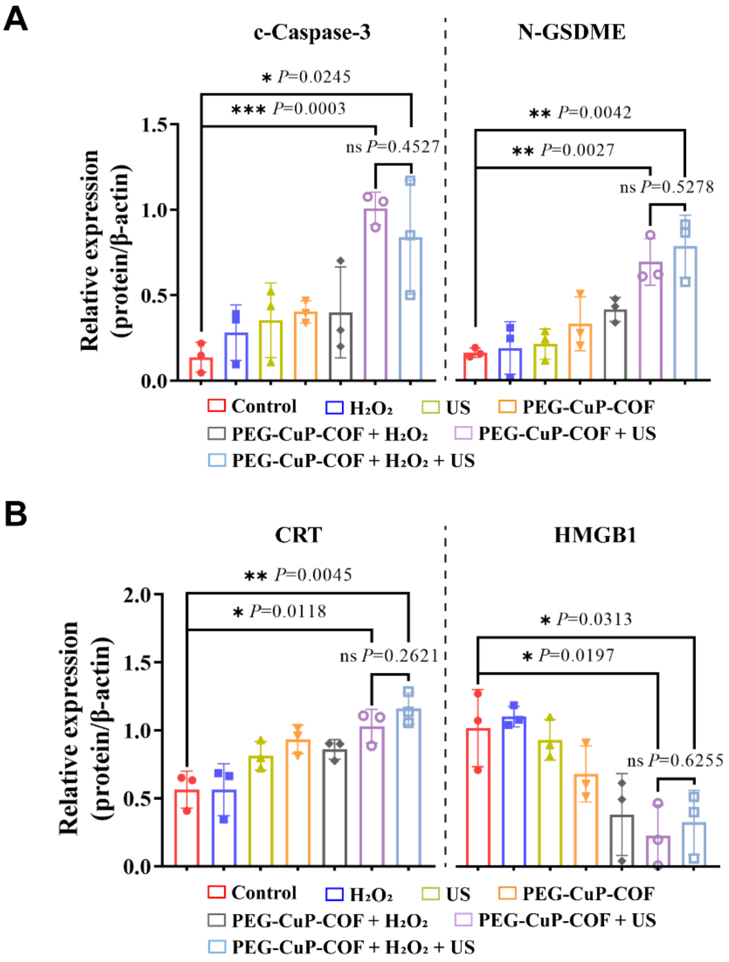

Figure S12. Quantitative analysis of A) c-Caspase-3, N-GSDME, B) CRT and HMGB1 based on the western blot result. Data are presented as Mean ± SD. $^{****}P < 0.0001$, $^{***}P < 0.001$, $^{**}P < 0.01$, $^{*}P < 0.05$, ns: no significance.

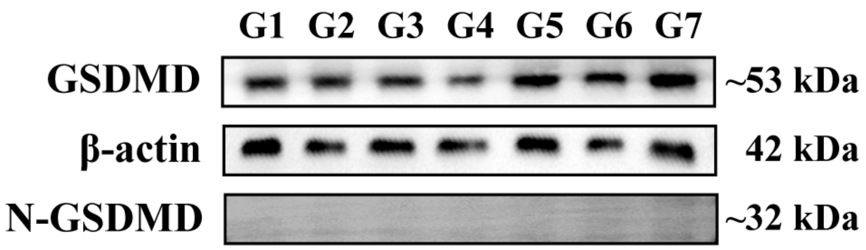

Figure S13. Western blot analysis of expression levels of GSDMD and N-GSDMD in RM-1 cells for various groups. G1: Control, G2: H_2O_2, G3: US only, G4: PEG-CuP-COF, G5: PEG-CuP-COF + H_2O_2, G6: PEG-CuP-COF + US, G7: PEG-CuP-COF + H_2O_2 + US.

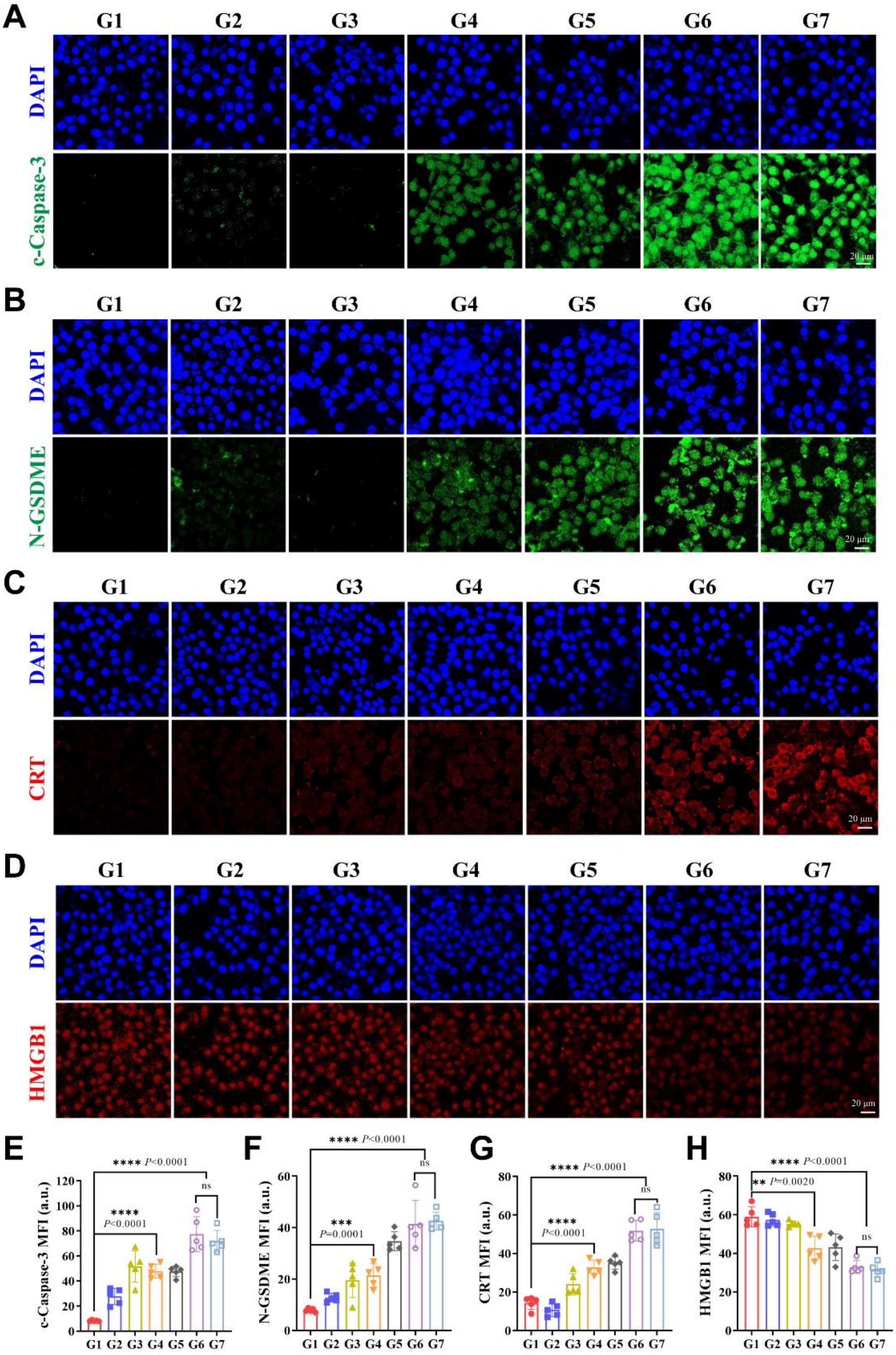

Figure S14. A-D) Immunofluorescence images and E-H) corresponding mean fluorescence intensity of c-Caspase-3, N-GSDME, CRT and HMGB1 in RM-1 cells for various groups. G1: Control, G2: H_2O_2, G3: US only, G4: PEG-CuP-COF, G5: PEG-CuP-COF + H_2O_2, G6: PEG-CuP-COF + US, G7: PEG-CuP-COF + H_2O_2 + US. Data are presented as Mean ± SD. $^{****}P < 0.0001$, $^{***}P < 0.001$, $^{**}P < 0.01$, $^{*}P < 0.05$, ns: no significance.

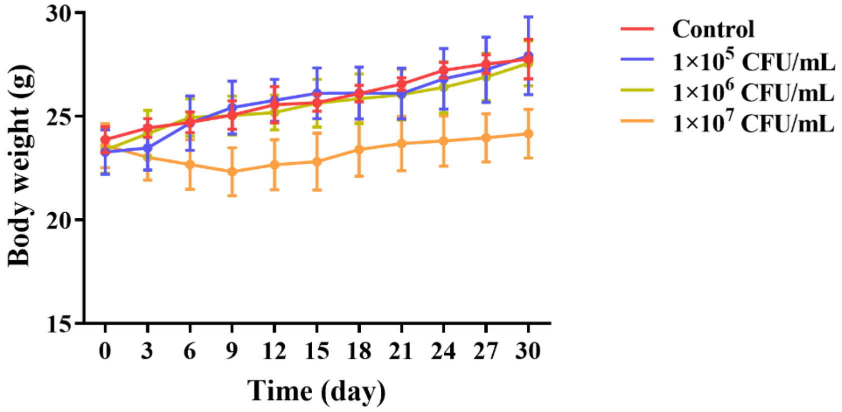

Figure S15. Body weight monitoring of mice during 30 days after injection of different doses of bacteria (200 μL per mouse). Control *i.e.* without any treatment. Data are presented as Mean ± SD.

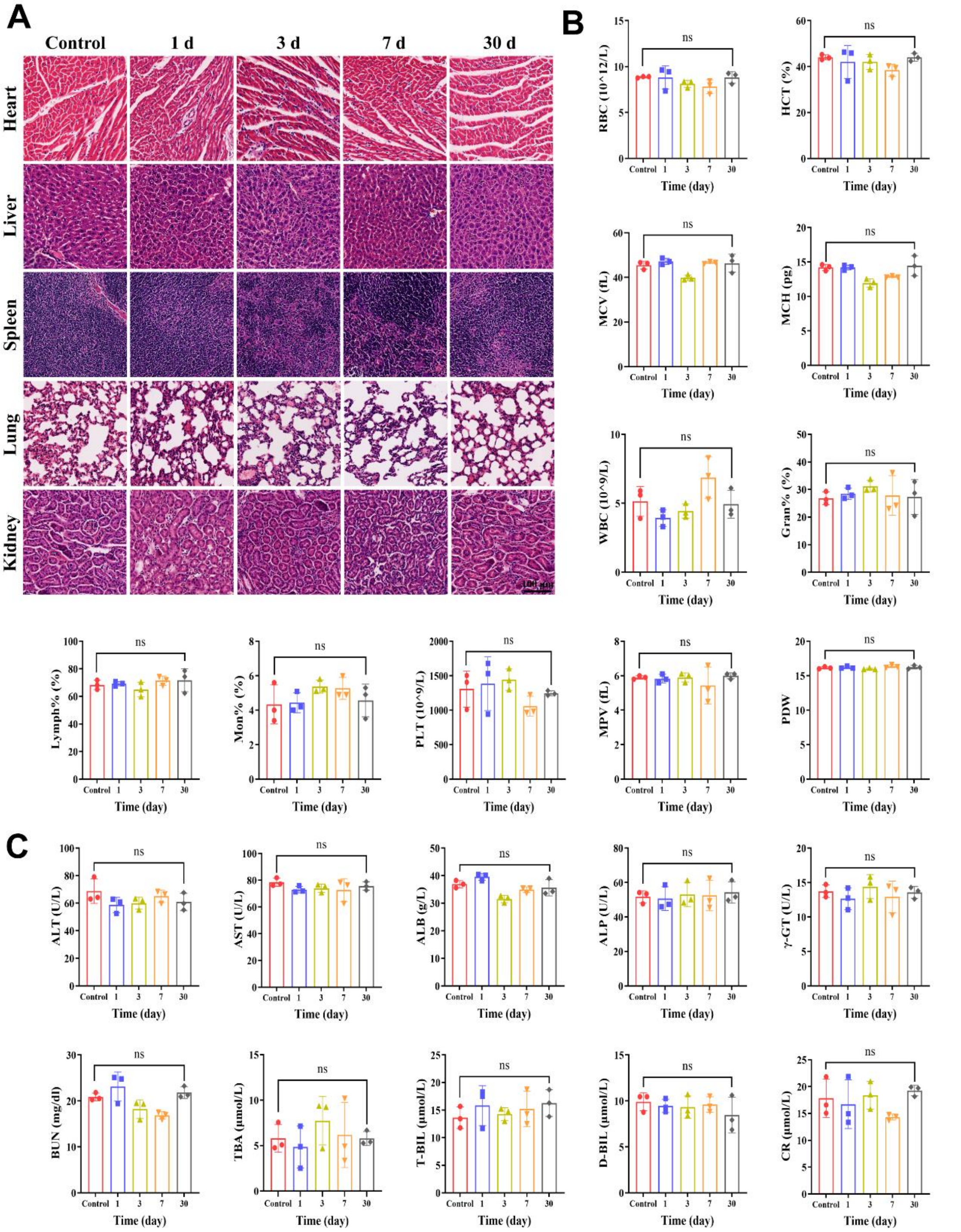

Figure S16. A) H&E-stained major organs sections, B) hematology and C) blood biochemical examination of the mice at 1, 3, 7 and 30 days after PEG-CuP-COF@ΔSt injection (200 μL per mouse, 1 mL PEG-CuP-COF@ΔSt contained 1×10^6 CFU ΔSt and 1.0 mg PEG-CuP-COF). Control *i.e.* without any treatment. Data are presented as Mean ± SD. $^{****}P < 0.0001$, $^{***}P < 0.001$, $^{**}P < 0.01$, $^{*}P < 0.05$, ns: no significance.

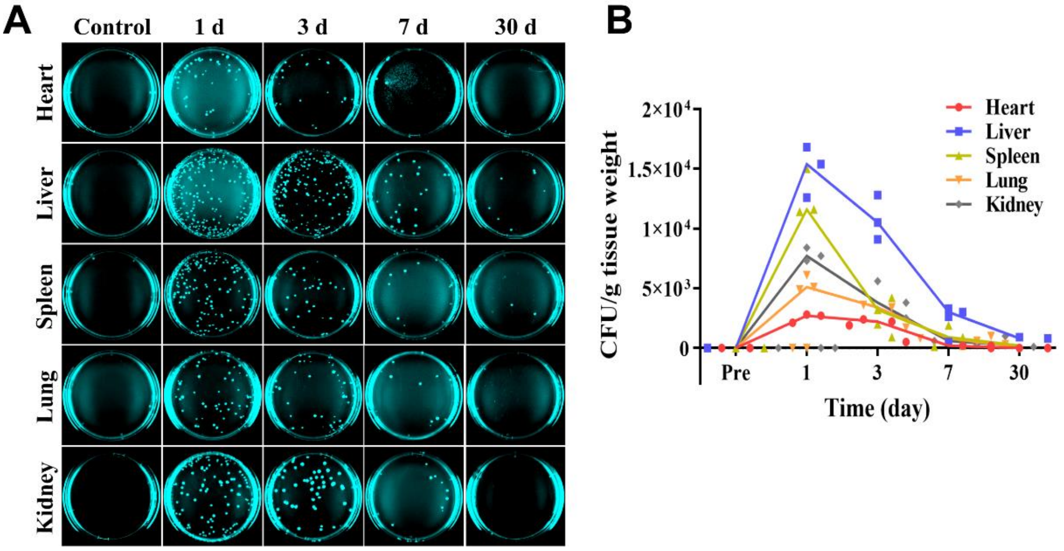

Figure S17. A) Representative photographs of LB agar plates and B) corresponding CFU quantification of bacterial colonization in major organs after PEG-CuP-COF@ΔSt injection in a month (1, 3, 7 and 30 days). Control *i.e.* without any treatment. Data are presented as Mean ± SD.

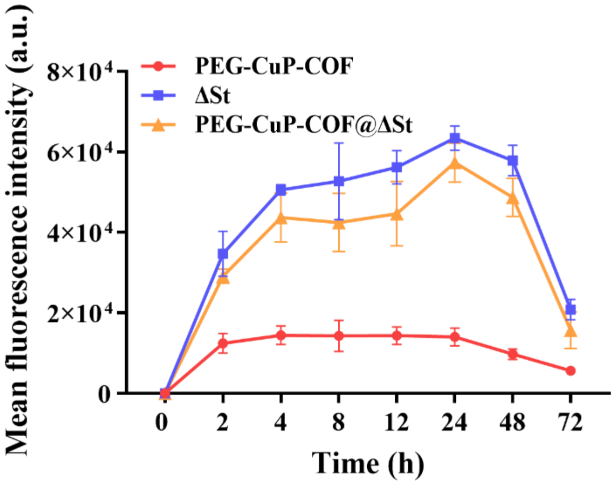

Figure S18. Tumor fluorescence intensity of Cy5.5-labeled PEG-CuP-COF, Cy5.5-labeled ΔSt and Cy5.5-labeled PEG-CuP-COF@ΔSt in mice. Data are presented as Mean ± SD.

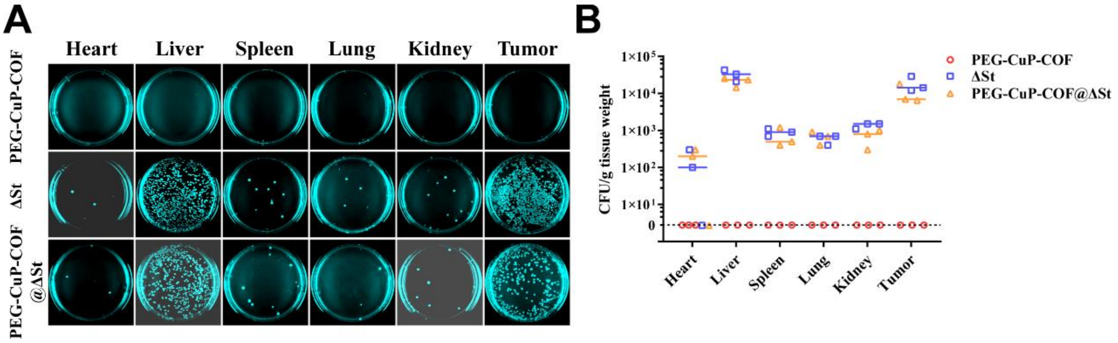

Figure S19. A) Representative photographs of LB agar plates and B) corresponding quantitative analysis of bacterial colonization in various organs and tumors of RM-1 tumor-bearing mice after injection of PEG-CuP-COF, ΔSt and PEG-CuP-COF@ΔSt. Data are presented as Mean ± SD.

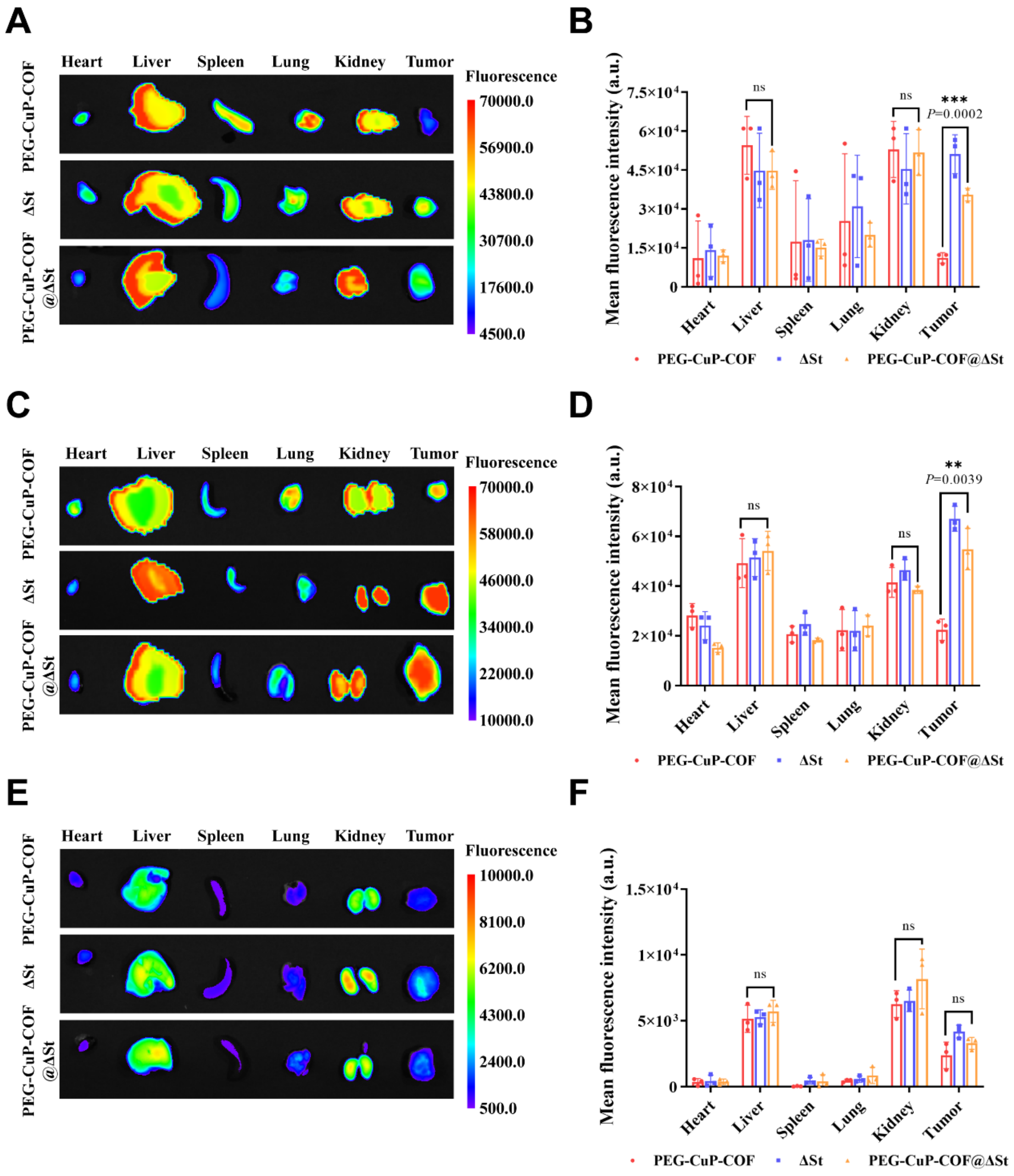

Figure S20. A) Fluorescence imaging and B) corresponding quantification of mean fluorescence intensity of major organs and tumors in 8-h post-injection. C) Fluorescence imaging and D) corresponding quantification of mean fluorescence intensity of major organs and tumors in 24-h post-injection. E) Fluorescence imaging and F) corresponding quantification of mean fluorescence intensity of major organs and tumors in 72-h post-injection. Data are presented as Mean ± SD. $^{****}P < 0.0001$, $^{***}P < 0.001$, $^{**}P < 0.01$, $^{*}P < 0.05$, ns: no significance.

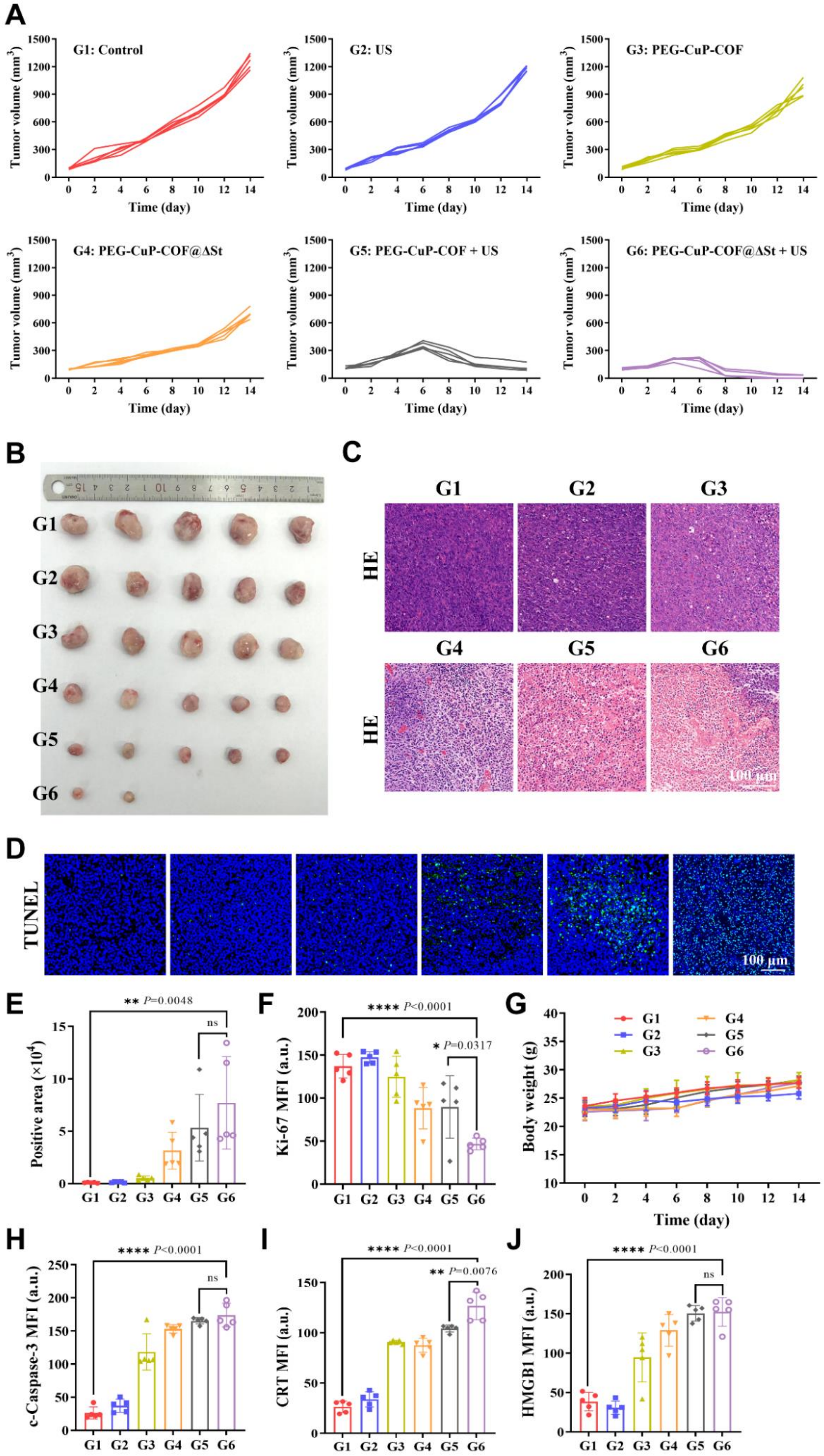
A
G1: Control
G2: US
G3: PEG-CuP-COF
G4: PEG-CuP-COF@ΔSt
G5: PEG-CuP-COF + US
G6: PEG-CuP-COF@ΔSt + US
Tumor volume (mm3)
Time (day)
B
C
HE
100 μm
D
TUNEL
E
Positive area (×10⁴)
** P=0.0048
ns
F
Ki-67 MFI (a.u.)
**** P<0.0001
* P=0.0317
G
Body weight (g)
H
c-Caspase-3 MFI (a.u.)
I
CRT MFI (a.u.)
** P=0.0076
J
HMGB1 MFI (a.u.)
G1 G2 G3 G4 G5 G6

Figure S21. A) Tumor growth curves in each group (*n* = 5). B) Digital images and C) H&E-stained primary tumor sections after different treatments. D) Immunofluorescence images and E) corresponding fluorescence intensity of TUNEL assay in primary tumor tissues after different treatments. F) Fluorescence intensity of Ki-67 in primary tumors after various treatments. G) Body weight of RM-1 tumor-bearing mice in each group. H) Fluorescence intensity of c-Caspase-3 in primary tumors after various treatments. I) Fluorescence intensity of CRT in primary tumors after various treatments. J) Fluorescence intensity of HMGB1 in primary tumors after various treatments. G1: Control, G2: US only, G3: PEG-CuP-COF, G4: PEG-CuP-COF@ΔSt, G5: PEG-CuP-COF + US, G6: PEG-CuP-COF@ΔSt + US. Data are presented as Mean ± SD. $^{****}P < 0.0001$, $^{***}P < 0.001$, $^{**}P < 0.01$, $^{*}P < 0.05$, ns: no significance.

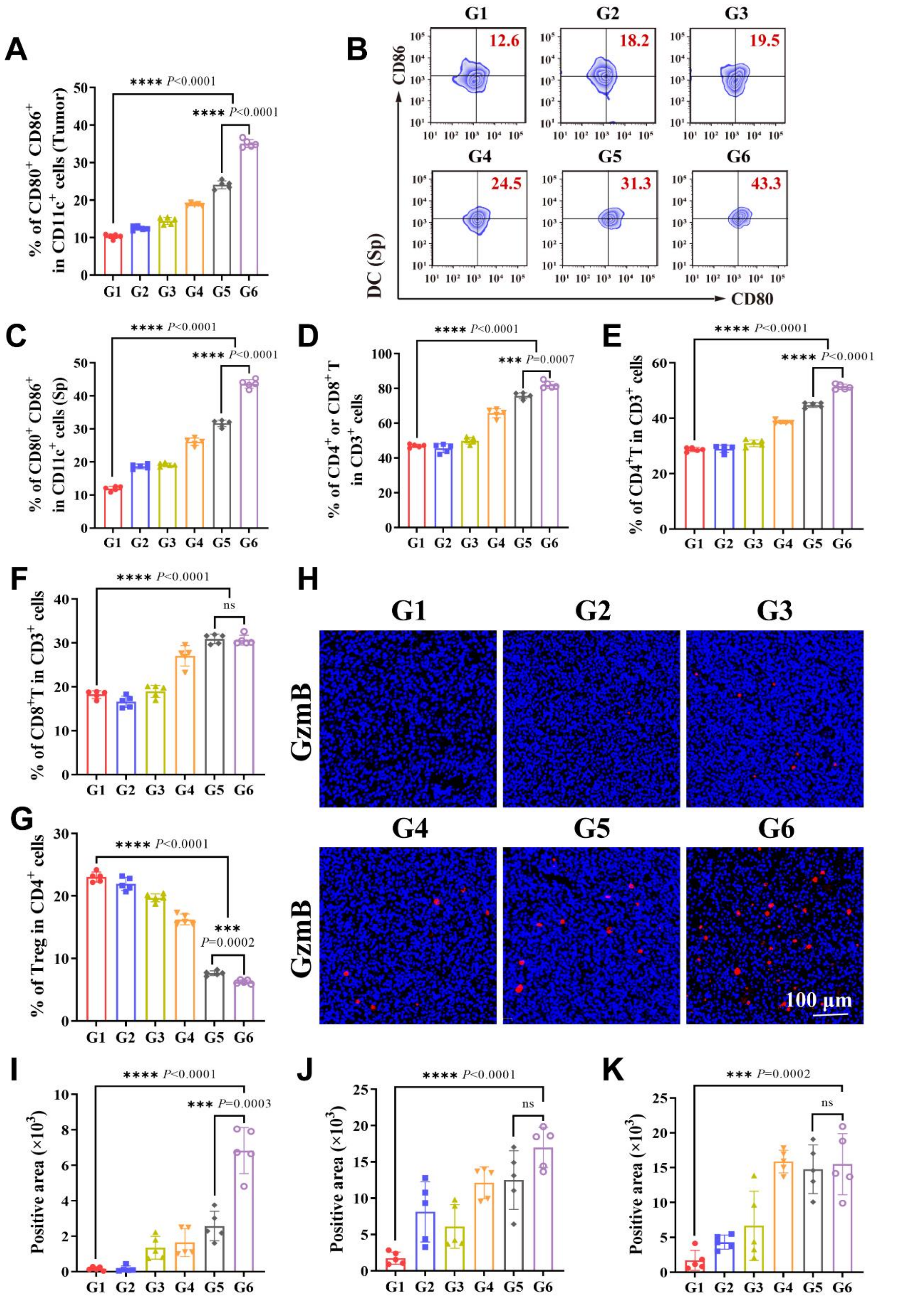

Figure S22. A) Quantitative analysis of mature DCs in tumor tissues after initial various treatments. B) Typical flow cytometry of mature DCs and C) corresponding quantitative analysis in the spleen (Sp) following initial various treatments. D-F) Quantitative analysis of $CD4^{+}$ and

CD8$^+$ T cells in the spleen after initial various treatments. G) Quantitative analysis of Tregs in tumor tissues after initial various treatments. H) Immunofluorescence images and I) corresponding positive area quantification of GzmB in tumor sections after initial various treatments. J) Positive area quantification of CD3$^+$CD4$^+$ and K) CD3$^+$CD8$^+$ T cells in primary tumor tissues. G1: Control, G2: US only, G3: PEG-CuP-COF, G4: PEG-CuP-COF@ΔSt, G5: PEG-CuP-COF + US, G6: PEG-CuP-COF@ΔSt + US. Data are presented as Mean ± SD. $^{****}P < 0.0001$, $^{***}P < 0.001$, $^{**}P < 0.01$, $^{*}P < 0.05$, ns: no significance.

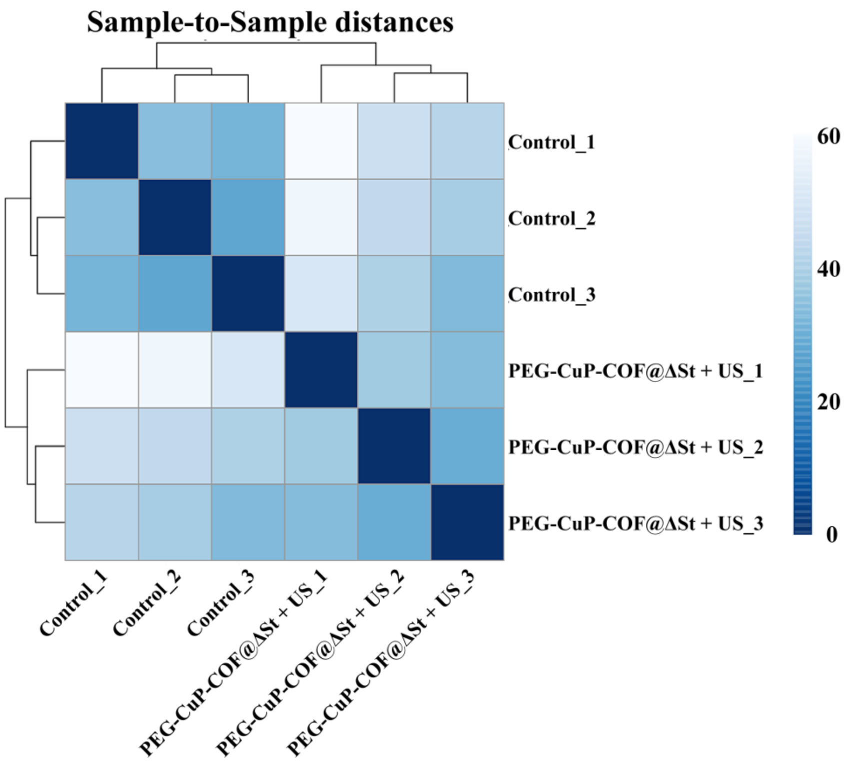

Figure S23. Unsupervised hierarchical clustering of the samples from the Control group and the PEG-CuP-COF@ΔSt + US group.

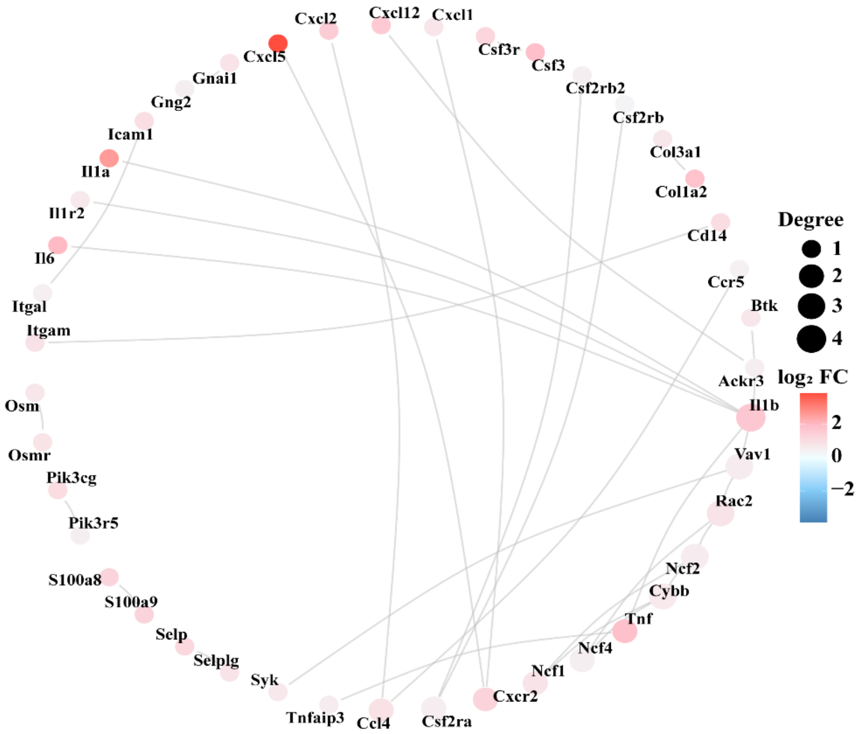

Figure S24. Protein-protein interaction network of these functional genes.

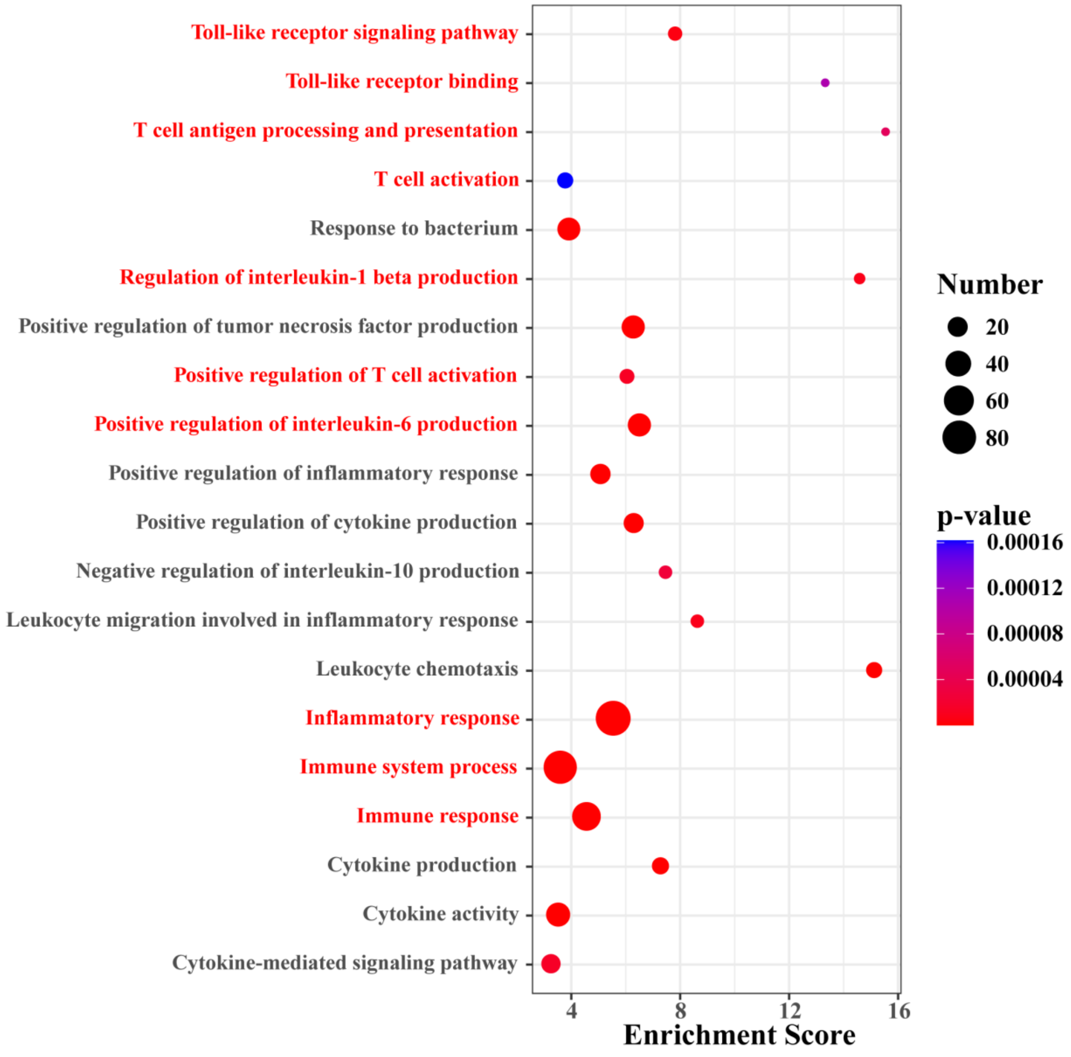

Figure S25. Results of gene ontology (GO) enrichment analysis of inflammation/immune-related DEGs (red).

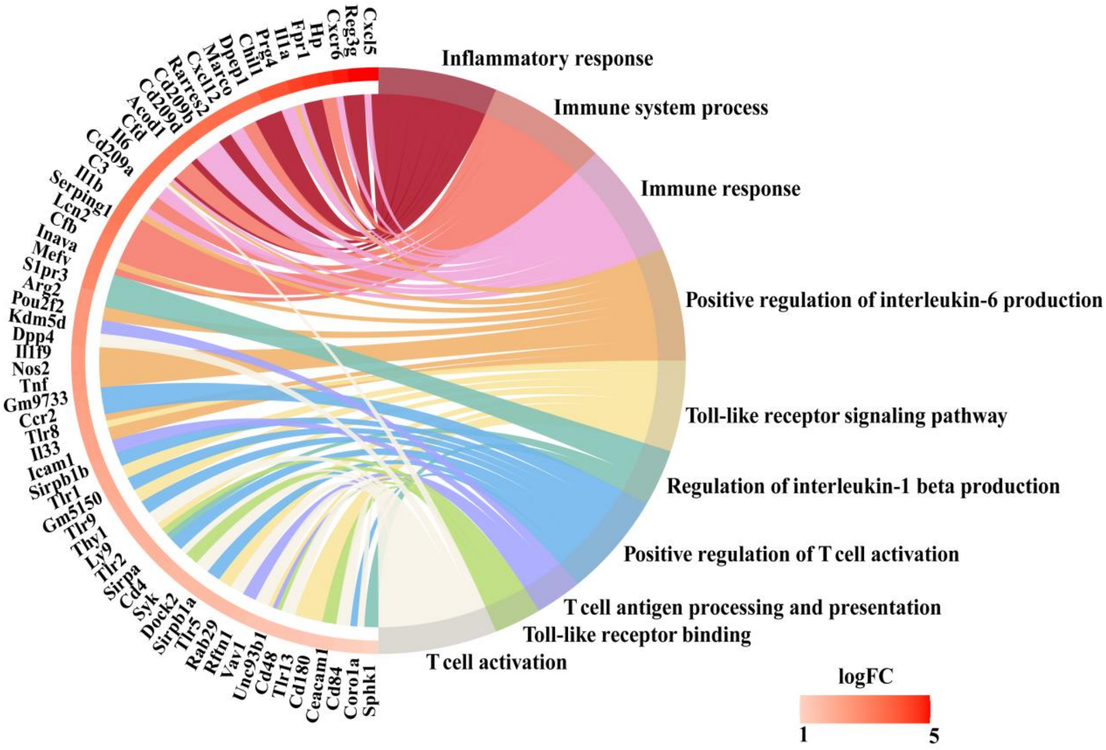

Figure S26. Circle diagram of gene ontology (GO) enrichment analysis of inflammation/immune-related DEGs.

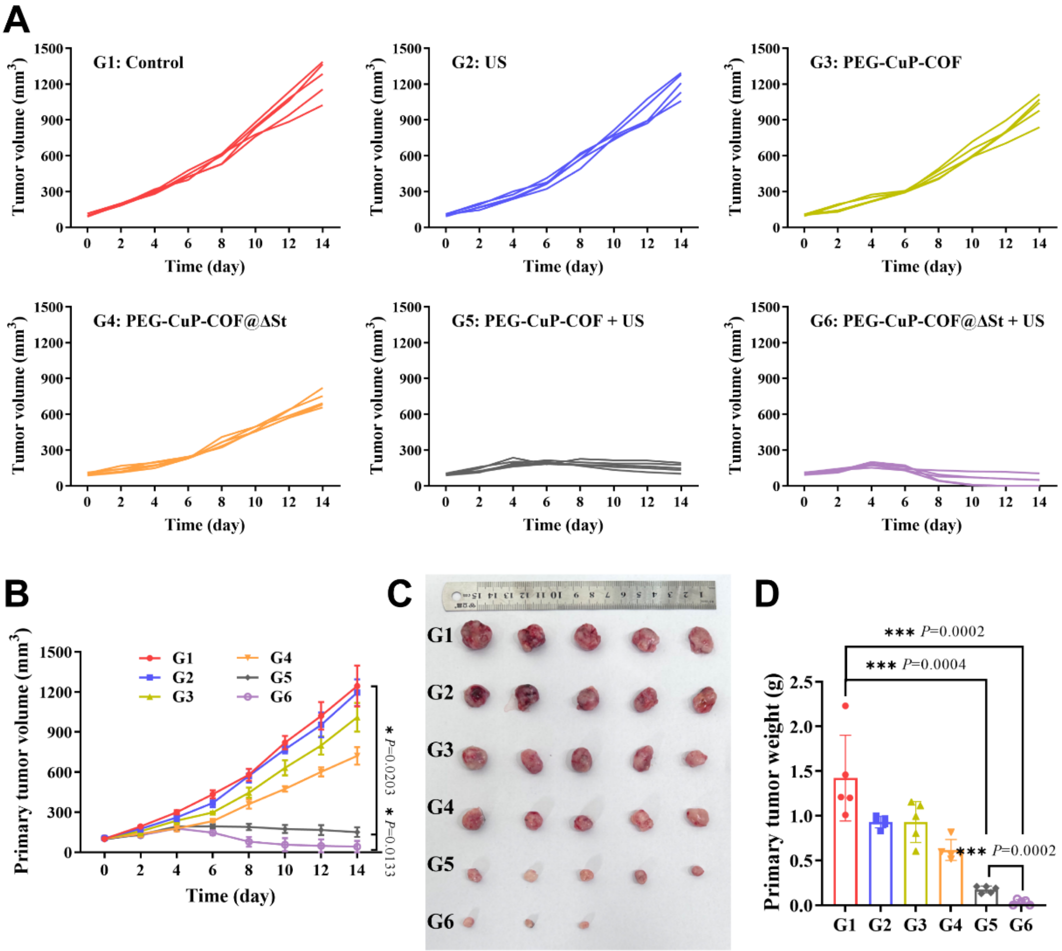

Figure S27. A) Primary tumor growth curves in each group (n = 5). B) Average tumor growth curves in various groups. C) Digital images and D) weight statistics of primary tumors in RM-1 tumor-bearing mice after different treatments. G1: Control, G2: US only, G3: PEG-CuP-COF, G4: PEG-CuP-COF@ΔSt, G5: PEG-CuP-COF + US, G6: PEG-CuP-COF@ΔSt + US. Data are presented as Mean ± SD. $^{****}P < 0.0001$, $^{***}P < 0.001$, $^{**}P < 0.01$, $^{*}P < 0.05$, ns: no significance.

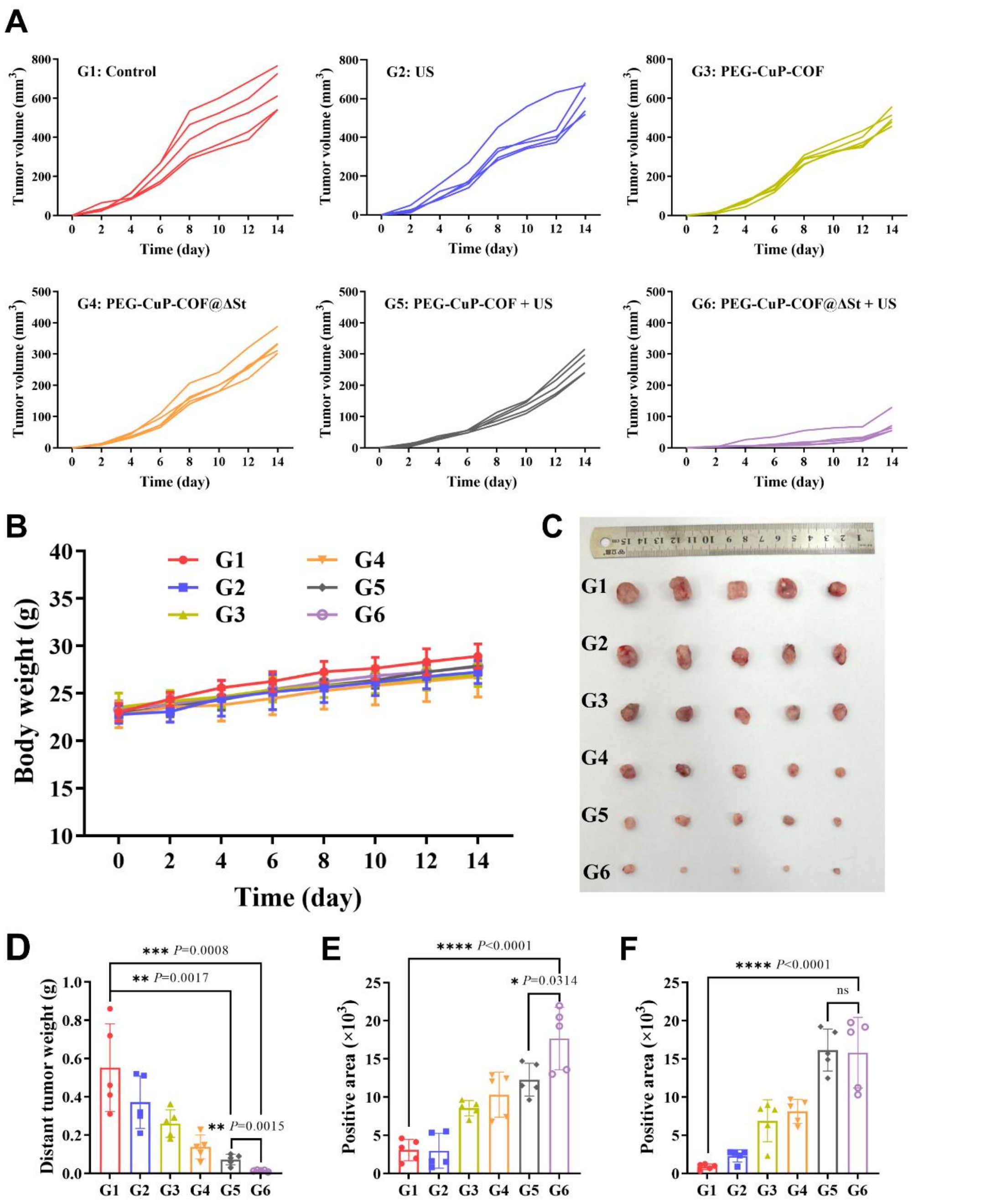

Figure S28. A) Distant tumor growth curves in each group (*n* = 5). B) Body weight in various groups. C) Digital images and D) weight statistics of distant tumors in RM-1 tumor-bearing mice after different treatments. E) Positive area quantification of $CD3^+CD4^+$ and F) $CD3^+CD8^+$ proliferating T cells in immunofluorescence images of distant tumor tissues after various treatments. G1: Control, G2: US only, G3: PEG-CuP-COF, G4: PEG-CuP-COF@ΔSt, G5: PEG-CuP-COF + US, G6: PEG-CuP-COF@ΔSt + US. Data are presented as Mean ± SD. $^{****}P$ < 0.0001, $^{***}P$ < 0.001, $^{**}P$ < 0.01, $^{*}P$ <0.05, ns: no significance.

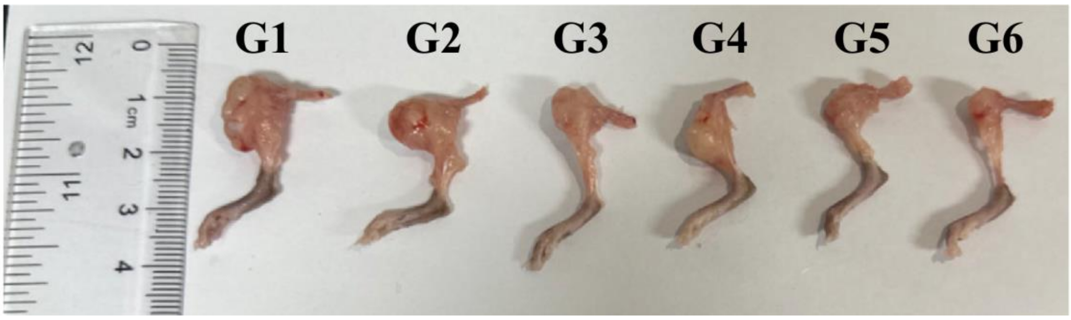

Figure S29. Digital image of metastatic bone tumors. G1: Control, G2: US only, G3: PEG-CuP-COF, G4: PEG-CuP-COF@ΔSt, G5: PEG-CuP-COF + US, G6: PEG-CuP-COF@ΔSt + US.

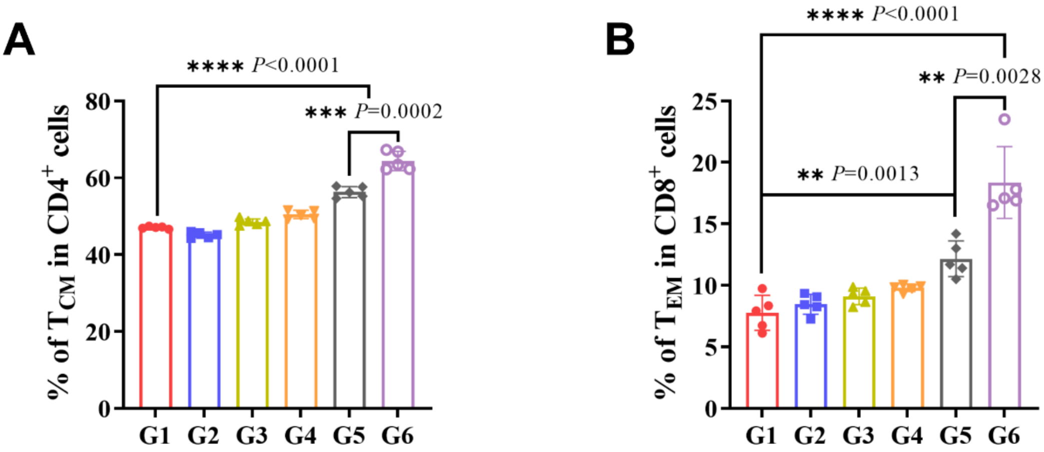

Figure S30. A) Quantitative analysis of T_{CM} and B) T_{EM} in the spleen after various treatments. G1: Control, G2: US only, G3: PEG-CuP-COF, G4: PEG-CuP-COF@ΔSt, G5: PEG-CuP-COF + US, G6: PEG-CuP-COF@ΔSt + US. Data are presented as Mean ± SD. $^{****}P < 0.0001$, $^{***}P < 0.001$, $^{**}P < 0.01$, $^{*}P < 0.05$, ns: no significance.

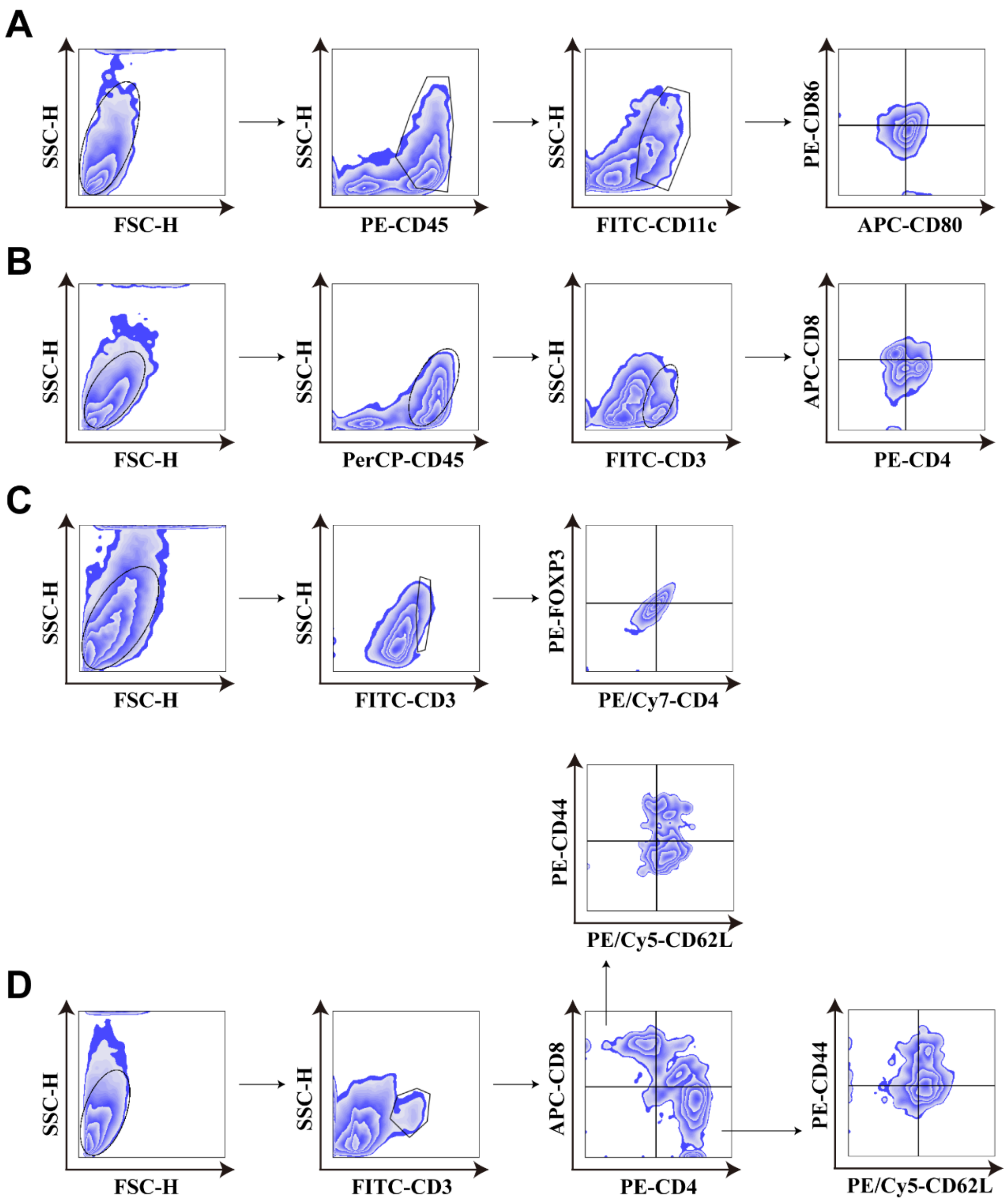

Figure S31. A) Gating strategies for isolating mature dendritic cells ($CD11c^+CD80^+CD86^+$, DCs) from tumor tissues. B) Gating strategies for isolating $CD4^+$ and $CD8^+$ T cells from spleen tissues. C) Gating strategies for isolating regulatory T cells ($CD3^+CD4^+Foxp3^+$, Tregs) from tumor tissues. D) Gating strategies for isolating central memory T cells ($CD3^+CD4^+CD44^+CD62L^+$, T_{CM}) and effector memory T cells ($CD3^+CD8^+CD44^+CD62L^-$, T_{EM}) from spleen tissues.
